# Selective Cleavage at CCA Ends and Anticodon Loops of tRNAs by Stress-Induced RNases

**DOI:** 10.3389/fmolb.2022.791094

**Published:** 2022-03-01

**Authors:** Yasutoshi Akiyama, Shawn M. Lyons, Marta M. Fay, Yoshihisa Tomioka, Takaaki Abe, Paul J. Anderson, Pavel Ivanov

**Affiliations:** ^1^ Laboratory of Oncology, Pharmacy Practice and Sciences, Tohoku University Graduate School of Pharmaceutical Sciences, Sendai, Japan; ^2^ Division of Rheumatology, Inflammation and Immunity, Brigham and Women’s Hospital, Boston, MA, United States; ^3^ Department of Medicine, Harvard Medical School, Boston, MA, United States; ^4^ Department of Biochemistry, Boston University School of Medicine, Boston, MA, United States; ^5^ The Genome Science Institute, Boston University School of Medicine, Boston, MA, United States; ^6^ Department of Medical Science, Tohoku University Graduate School of Biomedical Engineering, Sendai, Japan; ^7^ Department of Clinical Biology and Hormonal Regulation, Tohoku University Graduate School of Medicine, Sendai, Japan

**Keywords:** RNase A superfamily, angiogenin, tRNAs, CCA-terminus, stress response

## Abstract

Stress-induced tRNA cleavage has been implicated in various cellular processes, where tRNA fragments play diverse regulatory roles. Angiogenin (ANG), a member of the RNase A superfamily, induces cleavage of tRNAs resulting in the formation of tRNA-derived stress-induced RNAs (tiRNAs) that contribute to translational reprogramming aiming at cell survival. In addition to cleaving tRNA anticodon loops, ANG has been shown to cleave 3′-CCA termini of tRNAs *in vitro*, although it is not known whether this process occurs in cells. It has also been suggested that tiRNAs can be generated independently of ANG, although the role of other stress-induced RNases in tRNA cleavage is poorly understood. Using gene editing and biochemical approaches, we examined the involvement of ANG in stress-induced tRNA cleavage by focusing on its cleavage of CCA-termini as well as anticodon loops. We show that ANG is not responsible for CCA-deactivation under sodium arsenite (SA) treatment *in cellulo*, and although ANG treatment significantly increases 3′-tiRNA levels in cells, the majority of 3′-tiRNAs retain their 3′-CCA termini. Instead, other RNases can cleave CCA-termini in cells, although with low efficiency. Moreover, in the absence of ANG, other RNases are able to promote the production of tiRNAs in cells. Depletion of RNH1 (an endogenous inhibitor of RNase A superfamily) promotes constitutively-produced tiRNAs and CCA-deactivated tRNAs in cells. Interestingly, SA treatment in RNH1-depleted cells did not increase the amount of tiRNAs or CCA-deactivated tRNAs, suggesting that RNase A superfamily enzymes are largely responsible for SA-induced tRNA cleavage. We show that interplay between stress-induced RNases cause targeting tRNAs in a stress-specific manner *in cellulo*.

## Introduction

Angiogenin (ANG) is a secreted ribonuclease (RNase) that is a member of the RNase A superfamily (35% amino acid identity to RNase A) (Strydom et al., 1985). Although, ribonucleolytic activity of ANG is only a fraction of RNase A (Lee and Vallee 1989), most of its biological functions are critically dependent on its RNase activity (Shapiro et al., 1986; Shapiro and Vallee 1989; Curran et al., 1993). Although first identified as a tumor angiogenesis factor, ANG has been implicated in tumorigenesis, neurodegeneration, inflammation, pregnancy, and innate immunity [reviewed in (Sheng and Xu 2016)].

ANG is a stress-responsive RNase that is transcriptionally upregulated by stress (Pereira et al., 2010; Kishimoto et al., 2012) and modulates its RNase activity in response to stresses, especially oxidative stress [reviewed in (Lyons et al., 2017)]. Under optimal growth conditions, ANG is predominantly located in the nucleus where it stimulates rRNA biogenesis to promote cell growth and proliferation. Such localization is in contrast to other members of the RNase A superfamily, which are predominantly either cytoplasmic but endosome-compartmentalized or extracellular (Chao and Raines 2013). Moreover, different RNase A superfamily members are also different in their cell type- and tissue specificity. Although small proportion of ANG is found in the cytoplasm, it is held in an inactive state by RNH1, a universal inhibitor of ribonucleases belonging to the RNase A superfamily. Although RNH1 efficiently binds RNase A superfamily members with different affinities, molecular details of such interaction in *vivo* settings are unknown and may be regulated by post-translational modifications of RNases, such as ANG phosphorylation (Sarangdhar and Allam 2021). RNH1: RNase binding is suggested to neutralize enzymatic activities and cytotoxicity of RNases. In the absence of RNH1, even ANG (that is the least active enzymatically from all RNase A superfamily members) becomes cytotoxic (Thomas et al., 2018).

In response to different stress stimuli such as sodium arsenite (SA) treatment, ANG translocates into the cytoplasm from the nucleus, and dissociates from RNH1 (Pizzo et al., 2013). Under these conditions, ANG cleaves cytoplasmic tRNAs within their anticodon loops, generating two smaller RNA species called tRNA-derived stress-induced RNAs (tiRNAs) (Yamasaki et al., 2009) or tRNA halves (Fu et al., 2009). The functional role of 5′- and 3′-tiRNAs are just beginning to be determined. 5′-tiRNAs derived from tRNA^Ala^ and tRNA^Cys^ inhibit translation initiation by displacing the eIF4F complex from the cap structures of mRNAs (Ivanov et al., 2011) and facilitate the formation of stress granules (SGs), pro-survival cytoplasmic foci containing stalled pre-initiation ribosomal complexes (Emara et al., 2010; Kedersha et al., 2013; Anderson et al., 2015; Panas et al., 2016; Yue et al., 2021). In addition to translational repression, tiRNAs also promote survival by binding to Cytochrome c to prevent apoptosome formation (Saikia et al., 2014). Through these mechanisms, ANG promotes cell survival at low metabolic cost under stress conditions.

Although it has been reported that ANG efficiently cleaves 5S ribosomal RNA (Rybak and Vallee 1988) and other small RNAs such as snRNAs and tRNAs (Li et al., 2012) by *in vitro* experiments, its *in vivo* enzymatic activity appears to be specific and limited to tRNAs (Saxena et al., 1992), and promoter-associated RNAs, which modulate ribosomal DNA (rDNA) transcription (Hoang and Raines 2017). As for ANG-mediated tRNA cleavage, it has been suggested that ANG targets not only the anticodon but also the 3′-CCA terminus of tRNAs (Czech et al., 2013), the triplet that is added post-transcriptionally during tRNA maturation by the CCA-adding enzyme TRNT1. The same study also argued that oxidative stress induced by sodium arsenite (SA), known inducer of ANG-mediated tRNA cleavage, and also promotes CCA removal from tRNAs in live cells (Czech et al., 2013). Because ANG efficiently removes the terminal adenine residue from the 3′-CCA and recombinant TRNT1 re-adds the adenine *in vitro*, thus making tRNAs chargeable again, authors hypothesized that such reversible mechanism would also act as a stress-responsive switch to repress and reactivate translation at a low metabolic cost in live cells. Processing of CCA-termini of tRNAs is well-known mechanism for tRNA quality control in *E. coli* [reviewed in (Wellner et al., 2018)]. In addition, it has been recently reported that CCA-termini of tRNAs are shortened under nutritional stress in *Trypanosoma brucei* (Cristodero et al., 2021). Under stress condition, tRNAs are trimmed by nuclease LCCR4 resulting in translational repression, and repaired quickly once normal condition is restored (Cristodero et al., 2021). However, whether such mechanisms exist in human cells is still unclear because the data concerning ANG-mediated CCA-cleavage are based largely on *in vitro* experiments (Czech et al., 2013).

We have recently developed a novel, highly efficient method to generate ANG-mediated tiRNAs, called *in lysate* ANG digestion (Akiyama et al., 2021). Using this method, we showed that the specificity and efficiency of ANG-mediated cleavage largely depends on physiological conformation of RNAs. Under normal condition where RNAs are physiologically folded, ANG exclusively cleaves anticodon of tRNAs. Once the conformation of RNAs is disrupted by EDTA, other RNA species such as 7SK RNA, and 28S rRNAs are more actively cleaved by ANG into various length fragments. In contrast, interestingly, ANG-mediated tRNA cleavage was paradoxically abolished by EDTA, which suggests that ANG is optimized to cleave anticodon of physiologically folded tRNAs in the cell (Akiyama et al., 2021). Therefore, it is essential to investigate the specificity of ANG *in vivo* setting for clarifying the physiological function of ANG in the cell.

Although the pivotal roles of intracellular ANG are well studied, RNase A superfamily members are generally believed to function extracellularly [reviewed in (Koczera et al., 2016; Lu et al., 2018)]. Recently, two groups have reported the involvement of RNase A superfamily in the cleavage of extracellular RNAs including tRNAs, rRNAs, and Y RNAs (Nechooshtan et al., 2020; Tosar et al., 2020). However, intracellular roles of RNase A superfamily members except ANG are still largely unknown.

Here, we examined the involvement of ANG in stress-induced tRNA cleavage at both anticodon loops and 3′-CCA termini using SA. Using high-throughput RNA sequencing and biochemical approaches based on RNA ligation, we determined that although ANG targets CCA termini *in vitro*, it primarily targets anticodon loops, and not CCA termini in live cells. However, in agreement with data from the Ignatova lab (Czech et al., 2013), SA promotes cleavage of the CCA termini, although in an ANG-independent and oxidative stress-independent manners. This SA-induced cleavage is regulated by RNH1, a universal inhibitor of ribonucleases belonging to the RNase A superfamily, suggesting that other RNases related to ANG are also likely to be stress-responsive, and tRNA specific. Our data also suggest that in the genetic settings of ANG absence, other RNases are capable to generate tiRNAs providing “compensatory” tRNA cleavage in stressed cells.

## Materials and Methods

### Cell Culture and Treatment

The human osteosarcoma-derived cell lines U2OS were cultured at 37°C in a CO_2_ incubator in Dulbecco’s modified Eagle’s medium (DMEM) supplemented with 10% fetal bovine serum (FBS, Sigma) and 1% of penicillin/streptomycin (Sigma). For ANG treatment, U2OS cells were incubated with DMEM supplemented with 0.5 μg/ml recombinant human ANG for 1 h. Human recombinant ANG was prepared as reported previously (Wu et al., 2007). For sodium arsenite (SA, Sigma) treatment, cells were incubated in DMEM containing 500 µM of SA for 2 h.

### CCA-specific Ligation

Total RNAs were incubated at 37°C for 40 min in 20 mM Tris-HCl (pH 9.0) for deacylation of mature tRNAs, followed by purification with RNA Clean and Concentrator Kits (Zymo Research). 10 µg of deacylated total RNAs were incubated with 400 pmol of hairpin oligo (H-oligo) or double-strand oligo (ds-oligo) at 90°C for 2 min for denaturing. Denatured RNAs were then incubated in 5 mM Tris-HCl (pH 8.0), 500 µM EDTA, and 10 mM MgCl_2_ at 37°C for 15 min in 50-µl reaction volume for annealing. For RNA ligase 2-based ligation reaction, the annealed samples were incubated with 5 µl of 1x reaction buffer, 5 U (0.5 µl) of T4 RNA ligase 2 (Rnl_2_) (New England Biolabs) and 40 U (1 µl) of RNasin (Promega) at 37°C for 1 h, followed by overnight incubation at 4°C. For DNA ligase-based ligation, annealing was carried out in 16-µl reaction volume. Annealed samples were then incubated with 2 µl of 1x reaction buffer, 400 U (2 µl) of T4 DNA ligase (New England Biolabs) and 40 U (1 µl) of RNasin overnight at 16°C. The sequences of oligos are shown in Supplementary Table S1.

### CC-specific Ligation Combined With PNK Pre-treatment

The sequences of oligos for CC-specific ligation are shown in Supplementary Table S1. Before ligation reaction, samples were pre-treated with 10 U of T4 polynucleotide kinase (PNK) (New England Biolabs) at 37°C for 1 h according to the manufacturer’s instruction to remove 2′, 3′-cyclic phosphate residue. Ligation reaction was performed with Rnl_2_ as described in CCA-specific ligation section.

### Northern Blotting

Total RNA was extracted by using Trizol (Invitrogen). RNA was run on 10% or 15% TBE-urea gels (ThermoFisher Scientific), transferred to positively charged nylon membranes (Roche). The membranes were cross-linked by UV irradiation except for detecting 5′-tiRNA^Ile-AAT^, 5’tiRNA^Phe-GAA^, and 5′-tiRNA^Thr-AGT^. For detecting them, chemical cross-linking using 1-ethyl-3-(3-dimethylaminopropyl) carbodiimide (EDC, Sigma) was performed to improve the sensitivity as previously reported (Pall and Hamilton 2008). After cross-linking, the membranes were hybridized overnight at 40°C with digoxigenin (DIG)-labeled DNA probes in DIG Easy Hyb solution (Roche). After low stringency washes (washing twice with 2 × SSC/0.1% SDS at room temperature) and high stringency wash (washing once with 1 × SSC/0.1% SDS at 40°C), the membranes were blocked in blocking reagent (Roche) for 30 min at room temperature, probed with alkaline phosphatase-labeled anti-digoxigenin antibody (Roche) for 30 min, and washed with 1x TBS-T. Signals were visualized with CDP-Star ready-to-use (Roche) and detected using ChemiDoc imaging system (BioRad) according to the manufacturer’s instructions. Densitometry was performed using ImageJ software (NIH). Oligonucleotide probes were synthesized by IDT. DIG-labeled probes were prepared using the DIG Oligonucleotide tailing kit (2nd generation; Roche) according to the manufacturer’s instructions. The sequences of the probes are shown in Supplementary Table S2.

### Generation of ANG and RNH1 Knockout Cells

ANG knockout and RNH1 knockout U2OS cells were generated using CRISPR/Cas9-mediated gene editing as previously reported (Lyons et al., 2016). Briefly, oligonucleotides corresponding to a gRNA targeting the sequence 5′- TGG​TTT​GGC​ATC​ATA​GTG​CT-3′ in ANG and 5′-GAG​CCT​GGA​CAT​CCA​GTG​TG-3′ in RNH1 were designed using CRISPR Design software from the Zhang lab (crispr.mit.edu). Oligonucleotides were annealed and cloned into the pCas-Guide vector (Origene) according to the manufacturer’s protocol, and the resulting plasmid was co-transfected with pDonor-D09 (GeneCopoeia), which carries a puromycin resistance cassette, and into U2OS cells using Lipofectamine 2000 (Invitrogen). The following day, cells were selected with 1.5 μg/ml of puromycin for 24 h only, to lessen the likelihood of genomic incorporation of pDonor-D09. Cells were cloned by limiting dilution, and screened by western blot analysis using anti-ANG antibody (Santa Cruz, C-1) and anti-RNH1 antibody (Proteintech group, 10345-1-AP). Anti-beta-actin antibody (Proteintech group, 66009-1-Ig) was used as a loading control. Knockout was confirmed by genotyping and western blot analysis (Supplementary Figure S1).

### Knockdown of TRNT1

U2OS cells were transfected with siRNA against TRNT1 (ON-TARGETplus SMARTpool; GE Dharmacon, catalog #L-015850-00-0005) or Control siRNA (ON-TARGETplus Non-targeting siRNA #1; GE Dharmacon, catalog #D-001810-01-05) at a concentration of 40 nM using Lipofectamine 2000 (Invitrogen) according to the manufacturer’s protocol for reverse transfection. Ninety-six hours after transfection, cells were subjected to ANG treatment described above. Knockdown efficiency was checked by western blot analysis using anti-TRNT1 antibody (Novus, NBP1-86589). Anti-VDAC1 antibody (Santa Cruz, N-18) was used as a loading control.

### *In vitro* ANG Digestion

*In vitro* ANG digestion was performed according to the procedure described in (Czech et al., 2013) with slight modification. Briefly, total RNA in 30 mM HEPES (pH 7.0), 30 mM NaCl was heated at 90°C for 2 min and cooled down to room temperature. MgCl_2_ and BSA were added to final concentrations of 2 mM and 0.01%, respectively, and further incubated at 37°C for 5 min. Recombinant human ANG was added to a final concentration of 0.2 µM and incubated at 37°C for 4 h.

### RNA Sequencing Library Preparation for High-Throughput Sequencing

RNA-seq libraries were prepared from samples from three independent experiments. 10 µg of total RNA was run on 15% Urea-TBE gel, and 20–50 nt (tiRNA fraction) or 50–110 nt (tRNA fraction) was gel-purified using ZR small-RNA PAGE Recovery kit (Zymo Research). The purified RNAs were treated with calf intestinal alkaline phosphatase (New England Biolabs). After purification using Direct-zol RNA Microprep (Zymo Research), the RNAs were treated with T4 polynucleotide kinase (New England Biolabs), and then purified using Direct-zol RNA Microprep. Small RNA libraries were prepared using the TruSeq Small RNA library preparation kit (Illumina) according to the manufacturer’s protocol. Sequencing was performed on the Illumina platform (Molecular Biology Core Facility, Dana-Farber Cancer Institute, Boston, United States), and 75 bp single-end reads (tiRNA fraction) or 150 bp single-end reads (tRNA fraction) were generated.

### Calculation of the Proportion of Fragments That Have CCA or CC at Their 3′-Termini

For accurate mapping to the reference genome, we generated two sets of adapter-trimmed data using cutadapt (Martin 2011). In the first set, only the adapter sequence was trimmed (“Only Adapter-trimmed”), and in the second, the 3′-CCA or 3′-CC and adapter sequences were trimmed (“CCA-trimmed”) (Supplementary Figure S2B). Then “CCA-trimmed” data set was aligned to hg38 reference genome (obtained from UCSC Genome Browser (Karolchik et al., 2003)) using Bowtie (Langmead et al., 2009) with parameters "-k 1 --best -v 3″ which reports one best match with up to 3 mismatches. Up to 3 mismatches were allowed in order to prevent mapping failure for tRNA-derived reads with misincorporations due to modification sites. Reads that overlapped 3′-end (5 nt) of tRNA genes were extracted as “3′-end containing reads” using samtools (Li et al., 2009) (Supplementary Figure S2C). The reads with the same ID were extracted as FASTQ format files from “Only Adapter-trimmed” data using seqtk (https://github.com/lh3/seqtk). There are 109 tRNA genes and 22 tRNA genes ending with CCA and CC, respectively (Supplementary Figure S2D). Immature tRNAs (end-processed, but without CCA addition) derived from these genes also ends with CCA or CC. To distinguish these reads from CCA-added or CC-terminating reads, 631 tRNA genes were divided into 3 groups: 1) ending with CCA (109 genes), 2) ending with CC (22 genes), and 3) the others (500 genes). Then the number and the proportions of CCA-added or CC-terminating reads were calculated in each group (Supplementary Figure S2D). The annotation information of genomic (cytoplasmic) tRNA genes were obtained from UCSC Genome Browser (Karolchik et al., 2003). Genomic tRNA sequences were obtained from genomic tRNA database (gtRNAdb) (Chan and Lowe 2009). The sequence and annotation information of mitochondrial tRNA^Ser-GCT^ were obtained from the Mamit-tRNA database (Putz et al., 2007). The read counts were presented as reads per million mapped reads (PMMR).

### Quantitative PCR

First strand cDNA was synthesized using Transcriptor First Strand cDNA Synthesis Kit (Roche). Quantitative real-time PCR was performed using the TaqMan Gene Expression Assay (Applied Biosystems), according to the manufacturer’s instructions using a StepOnePlus Real Time PCR System (Applied Biosystems). The relative ratios of mRNA levels were calculated using the ∆∆Ct method normalized with GAPDH Ct value. The TaqMan probes used in this study are as follows: RNASE1 (Assay ID: Hs01850125_s1); RNASE4 (Assay ID: Hs00268002_s1); ANG (Assay ID: Hs02379000_s1); and GAPDH (Assay ID: Hs02758991_g1).

### Statistical Analyses

Comparisons between groups were performed using one-way ANOVA or an unpaired Student’s t-test as appropriate. The Dunnett’s test was applied to compare experimental groups with control. The Tukey–Kramer test was used for multiple comparison.

## Results

### Angiogenin Does Not Target CCA Ends of tRNAs *in Cellulo*

ANG is reported to target tRNAs in anticodon loops and 3′-CCA termini *in vitro* and in response to oxidative stress induced by SA in cells (Czech et al., 2013). To clarify the effect of ANG on tRNA 3′-CCA termini *in cellulo*, we determined whether ANG treatment changed the proportion of CCA-added tRNAs or 3′-tiRNAs using high-throughput RNA sequencing (RNA-seq). Because SA treatment induces pleiotropic effects on cell physiology that may or not be related to its induction of ANG translocation or/and induction of oxidative stress, we adapted previously described cellular model based on recombinant ANG treatment (Shapiro et al., 1988; Shapiro and Vallee 1992; Wu et al., 2007; Yamasaki et al., 2009; Emara et al., 2010; Saikia et al., 2014; Yu et al., 2017). Since it has been reported that ANG cleaves within the 3′-CCA termini between the C and A *in vitro* (Czech et al., 2013), if a similar reaction occurs *in cellulo*, cleaved tRNAs should bear CC-termini. Therefore, we calculated the number and proportion of tRNAs or 3′-tiRNAs with CC as well as with CCA at their 3′-termini.

**FIGURE 1:**
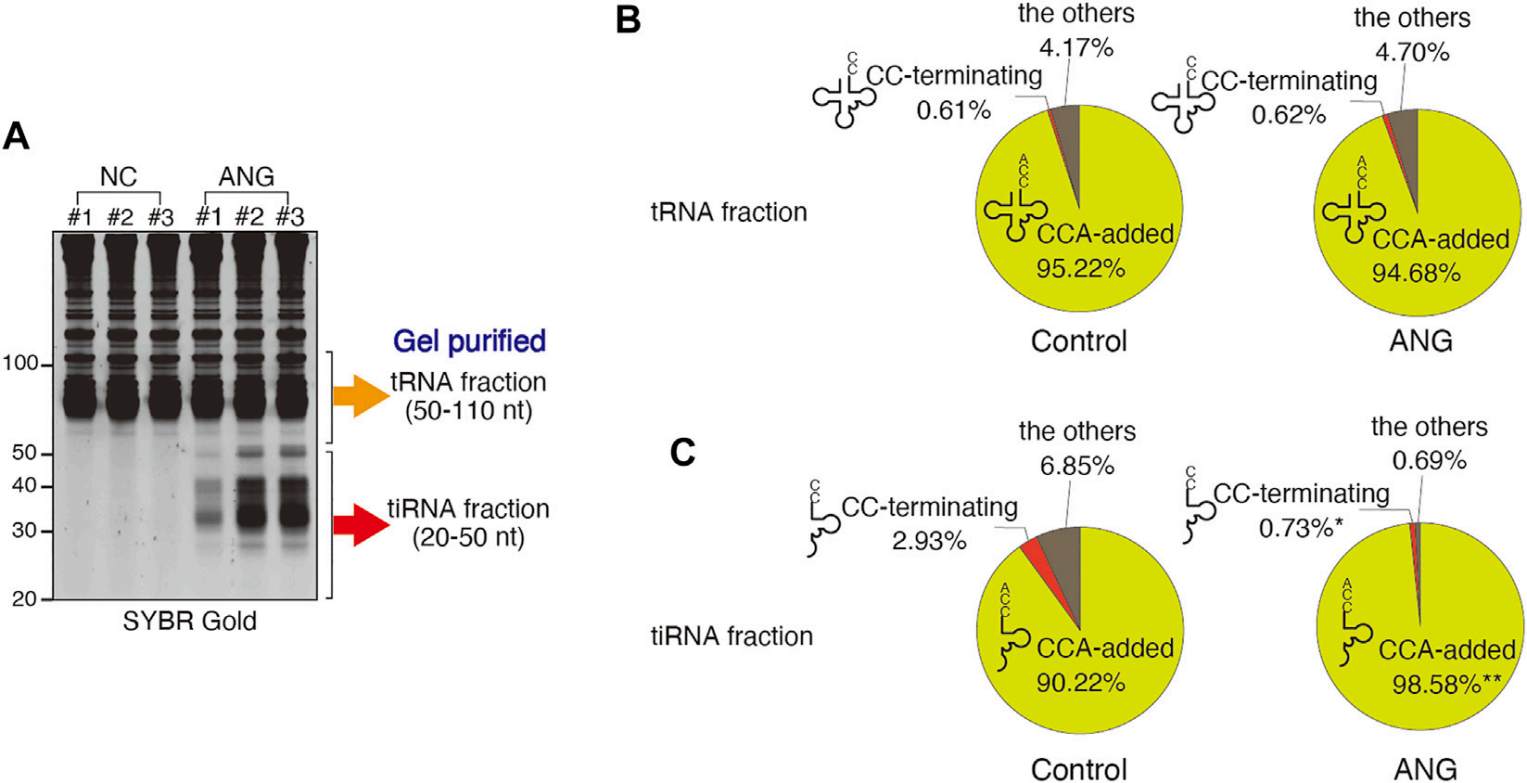
Effect of ANG treatment (at 0.5 μg/ml for 1 h) on the proportions of the tRNA-derived reads that have 3′-CCA or 3′-CC termini. **(A)** ANG-mediated tiRNA production in U2OS cells detected by SYBR Gold staining. The libraries were generated from gel-purified tRNA fraction (50–110 nt) or tiRNA fraction (20–50 nt). **(B)** Distribution of reads (in %) in tRNA fraction. Raw data can be found in Supplementary Table S3A. **(C)** Distribution of reads (in %) in tiRNA fraction. Raw data can be found in Supplementary Table S3B. The results are shown as pie charts. *: *p* < 0.05 VS Control, **: *p* < 0.01 VS Control.

Recombinant ANG added to cell culture media is rapidly internalized and cleaves mature cytoplasmic tRNAs (Hu et al., 2000; Yamasaki et al., 2009). We verified that ANG treatment induced tiRNA production (Figure 1A, 3 biological replicates). Next, we generated RNA sequencing libraries from gel-purified fractions (tRNA fraction and tiRNA fraction, Figure 1A). ANG-cleaved 5′-tRNA products start with 5′-monophosphates and end with 2′, 3′-cyclic phosphates, whereas 3′-tRNA products start with a hydroxyl residue and end with a hydroxyl residue (Yang 2011). Since traditional RNA-seq libraries are designed to efficiently capture 5′-monophosphate/3′-hydroxyl groups of RNAs, ANG-derived RNA fragments are not optimal for library construction. Therefore, we treated total RNA samples with calf intestinal phosphatase (CIP) and T4 polynucleotide kinase (PNK) prior to library preparation in order to convert tiRNAs to proper substrates for library preparation (Supplementary Figure S2A). Using Illumina sequencing, we then generated 75 bp single-end reads (tiRNA fraction) or 150 bp single-end reads (tRNA fraction) and calculated the proportion of these reads that end with CCA or CC (as described in Supplementary Figure S2 and Supplementary Table S3).

In the tRNA fraction, the total number of reads (mainly mature tRNAs) was slightly but significantly decreased by ANG treatment (188,990 PMMR (per million mapped reads) and 164,810 PMMR for control and ANG, respectively) (Supplementary Table S3A). CCA-terminating tRNAs were also slightly decreased by ANG treatment (179,947 PMMR and 156,044 PMMR for control and ANG, respectively). However, the number of CC-terminating tRNAs was not increased by ANG treatment (1,170 PMMR and 1,020 PMMR for control and ANG, respectively, Supplementary Table S3A), suggesting that the decrease of CCA-added tRNAs was not due to the ANG-mediated cleavage of 3′-CCA termini, but due to the decrease of full length tRNAs by ANG-mediated tiRNA production by targeting anticodon loops. The proportion of tRNAs with CCA did not change significantly following ANG treatment, 95.22% for control, and 94.68% for ANG (Figure 1B; Supplementary Table S3A). The proportion of CC-terminating tRNAs was small and not altered by ANG treatment, 0.61% for control and 0.62% for ANG (Figure 1B; Supplementary Table S3A). These data demonstrate that ANG does not efficiently target tRNA 3′-CCA termini *in cellulo*, as most the tRNAs remain intact and unchanged after ANG treatment.

It has been reported that 3′-CCA cleavage by ANG precedes tiRNA production *in vitro* (Czech et al., 2013). If this occurs *in vivo*, most 3′-tiRNAs would exist as CCA-cleaved form. We examined the 3′-termini of 3′-tiRNAs after treating cells with ANG (Supplementary Table S3B; Figure 1C). ANG treatment results in the production of 5′-tiRNAs from all tRNA species (represented by 22 amino acids, Supplementary Figure S3), thus reassuring that potential 3′-CCA cleavage step has been already occurred (Czech et al., 2013). The total number of reads (mostly 3′-tiRNAs) was significantly increased by ANG treatment (from 38,375 PMMR in control to 240,173 PMMR in ANG, ∼6.3-fold increase) consistent with previous reports (Fu et al., 2009; Yamasaki et al., 2009; Saikia et al., 2012). Further analysis indicated that most 3′-tiRNAs (98.58%) have CCA at their 3′-termini (Figure 1C; Supplementary Table S3B), which is consistent with the previous report (Su et al., 2019). Furthermore, the number of CC-terminating 3′-tiRNAs was not significantly increased by ANG treatment (1,151 PMMR and 1,765 PMMR for control and ANG, respectively). On the other hand, %CC was significantly decreased by ANG treatment (2.93% in control to 0.73% in ANG) due to the marked increase of CCA-added (i.e., intact) 3′-tiRNAs (Figure 1C).

### TRNT1 Does Not Repair CCA-Ends *in Cellulo* After ANG Cleavage

**FIGURE 2:**
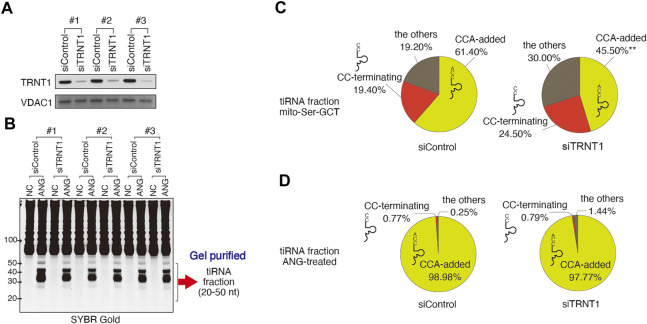
Effect of TRNT1 depletion on the proportion of tRNA-derived reads with 3′-CCA termini. **(A)** Knockdown efficiency of TRNT1 (siTRNT1, 96 h post-transfection) was evaluated by Western blot using TRNT1-specific antibody. Control siRNA (siControl) was used as control. VDAC1 was used as a loading control. Three biological replicates are shown. **(B)** SYBR Gold staining of total RNA prepared from cells treated with control or TRNT1-specific siRNAs, and left untreated (NC) or treated with recombinant ANG (ANG). tiRNA fraction (20–50 nt) was gel-purified and used for library preparation. **(C,D)** Effect of TRNT1 knockdown on the proportions of 3′-tiRNAs that have 3′-CCA or 3′-CC termini. **(C)** Distribution of reads (in %) in mitochondrial tRNA^Ser−GCT^ and **(D)** nuclear-encoded tRNAs. Raw data can be found in Supplementary Table S4.

*In vitro*, TRNA Nucleotidyl Transferase 1 (TRNT1) can repair CCA ends after ANG-mediated cleavage (Czech et al., 2013). In order to assess whether a similar mechanism occurs *in cellulo*, we examined whether the CCA-adding enzyme TRNT1 is involved in CCA repair following ANG treatment. To do so, we used TRNT1 depletion approach using siRNA targeting TRNT1. We reproducibly achieved an approximately 85% knockdown of TRNT1 protein (Figure 2A), which mimics previously reported hypomorphic phenotype of TRNT1 deficiency (Sasarman et al., 2015). RNAi mediated knockdown of TRNT1 caused no detectable difference in ANG-mediated tiRNA production (Figure 2B). Under these conditions, we generated RNA-seq libraries using gel-purified tiRNA fractions. To further confirm that TRNT1 knockdown affected TRNT1 activity, we calculated %CCA of mitochondrial tRNA^Ser-GCT^, because mitochondrial tRNA^Ser-GCT^ is most susceptible to TRNT1 hypofunction (Sasarman et al., 2015). In patients with TRNT1 hypomorphic mutations, CCA addition to mitochondrial tRNA^Ser-GCT^ is significantly affected (Sasarman et al., 2015). As expected, the total number of 3′-fragment reads derived from mitochondrial tRNA^Ser-GCT^ was significantly decreased by ANG treatment (59.0 PMMR and 21.4 PMMR for siControl-NC and siControl-ANG, respectively. *p* = 0.0068), because of the marked increase of tiRNAs derived from cytoplasmic tRNAs. However, ANG treatment did not induce significant cleavage of mitochondrial tRNA^Ser-GCT^ (Figure 2C; Supplementary Table S4A). In the non-treated samples (NC column in Supplementary Table S4A), the number of CCA-added 3′-fragments was significantly decreased by TRNT1 knockdown (36.6 PMMR and 17.5 PMMR for NC-siControl and NC-siTRNT1, respectively, *p* = 0.0409). The proportion of CCA-added 3′-fragments was also significantly decreased by TRNT1 knockdown (61.4 and 45.5% for NC-siControl and NC-siTRNT1, respectively. *p* = 0.0066). These data demonstrate that TRNT1 knockdown was effective enough to have a functional impact on CCA addition.

We then calculated the proportion of CCA-added and CC-terminating 3′-tiRNAs derived from cytoplasmic tRNAs in control and TRNT1 knockdown cells treated or not treated with ANG (Figure 2D; Supplementary Table S4B). As expected, ANG treatment increased levels of 3′-tiRNAs significantly both in control and TRNT1 knockdown cells. However, the percentage of CCA-terminating cytoplasmic 3′-tiRNAs did not change significantly following TRNT1 knockdown (98.98 and 97.77% for siControl and siTRNT1, respectively; *p* = 0.9731). In addition, the percentage of CC-terminating 3'-tiRNAs was not affected by TRNT1 knockdown in ANG-treated cells (0.77 and 0.79% for siControl and siTRNT1, respectively. *p* = 0.9998) (Figure 2D; Supplementary Table S4B). These results show that TRNT1 does not play a role in regulating ANG-dependent cleavage of tRNAs *in cellulo*.

### Probing Integrity of tRNAs’ CCA-Ends *in Cellulo* Using CCA-specific Ligation Method

**FIGURE 3:**
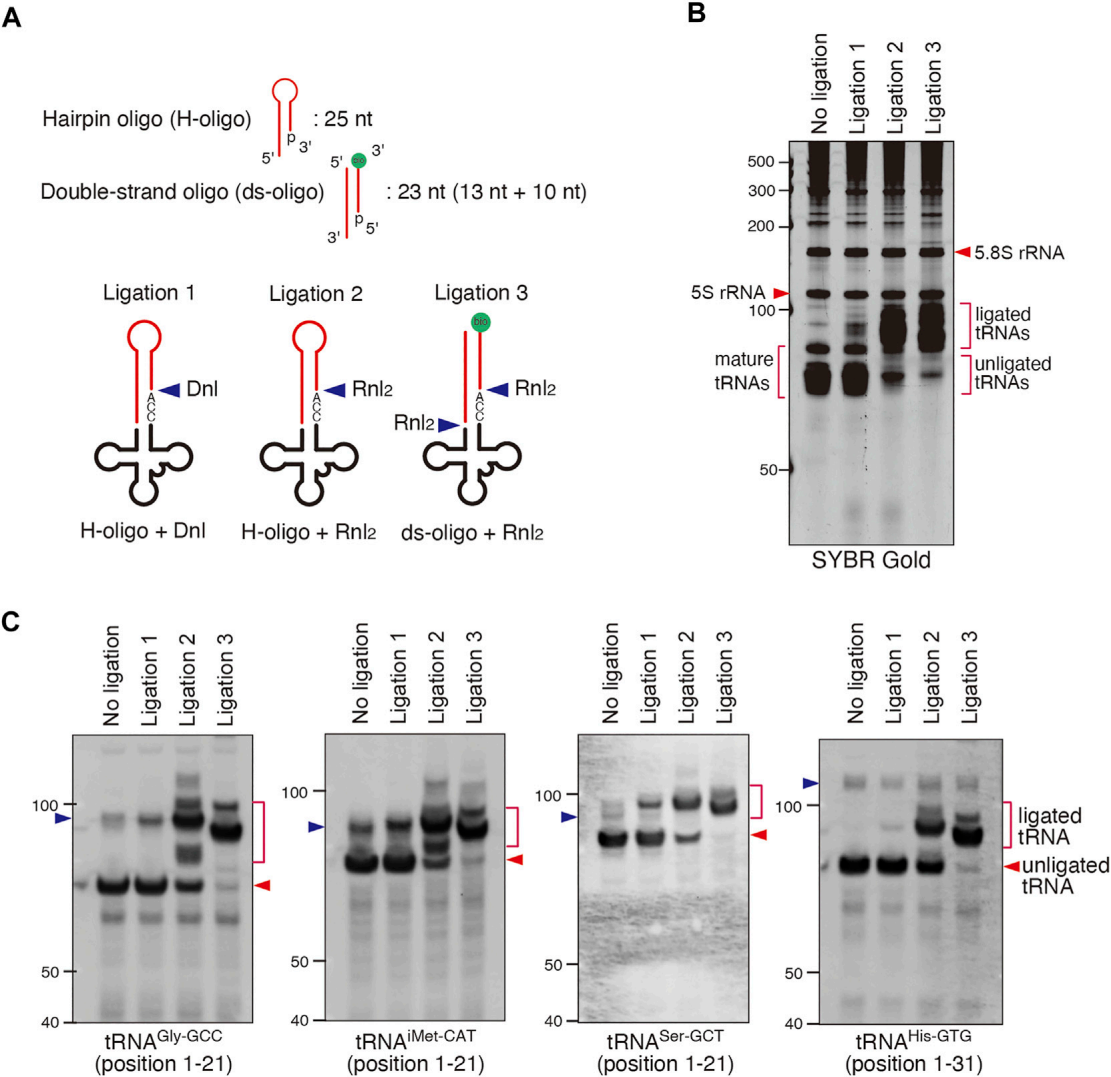
Validation of CCA-specific ligation methods. **(A)** Schema for three CCA-specific ligation methods. Dnl: T4 DNA ligase, Rnl_2_: T4 RNA ligase 2, bio: biotin. **(B,C)** The method using double-strand oligo and Rnl_2_ has the best ligation efficiency. **(B)** SYBR Gold staining and **(C)** Northern blotting for CCA-specific ligation products (ligated tRNA). The blue arrowheads indicate the bands for pre-tRNAs.

Since tRNA sequencing approaches have own limitations, we complemented our findings by alternative approaches. We examined the effect of ANG on tRNAs’ CCA-ends by biochemical approaches based on ligation of oligos specific to tRNAs possessing intact 3′-CCA termini. In this method named “CCA-specific ligation”, oligos which specifically anneal tRNAs possessing intact 3′-CCA termini are ligated resulting in longer ligated tRNA products (Figure 3A). First, we optimized the condition of ligation reaction using DNA ligase (Dnl) or RNA Ligase 2 (Rnl_2_) since the ligation efficiency can significantly differ depending on the combination of oligos and ligases (Bullard and Bowater 2006). In this method, CCA-intact tRNAs and CCA-deactivated tRNAs would be detected as longer ligation products (ligated tRNAs) and unligated tRNAs, respectively. Therefore, if ligation efficiency was low, it would be impossible to distinguish between CCA-deactivated tRNAs and unreacted tRNAs just due to low ligation efficiency. We also chose two substrates that were previously used to study the integrity of CCA ends, namely hairpin oligo (Dittmar et al., 2006; Czech et al., 2013) and double-stranded oligo [ds-oligo (Shigematsu et al., 2017)] (Figure 3A). As shown in Figures 3B,C, the combination of the Rnl_2_ ligase with ds-oligo resulted in the most efficient capture and ligation of mature CCA-intact tRNAs as judged by total tRNA shift by SYBR Gold staining (Figure 3B) and by the northern blotting against individual tRNA species (Figure 3C). Under this condition, most of mature tRNAs were ligated to ds-oligo and detected as “ligated tRNAs”, suggesting that almost all tRNAs were CCA-intact under this condition (Figure 3B). On the other hand, “Ligation 1″ method (combination of Dnl and hairpin oligo) showed the lowest ligation efficiency. Under this condition, most of mature tRNAs still remained as unligated tRNAs although most of them must have intact 3′-CCA termini. We conclude that “Ligation 3″ method is the best way to detect CCA-deactivated tRNAs.

**FIGURE 4:**
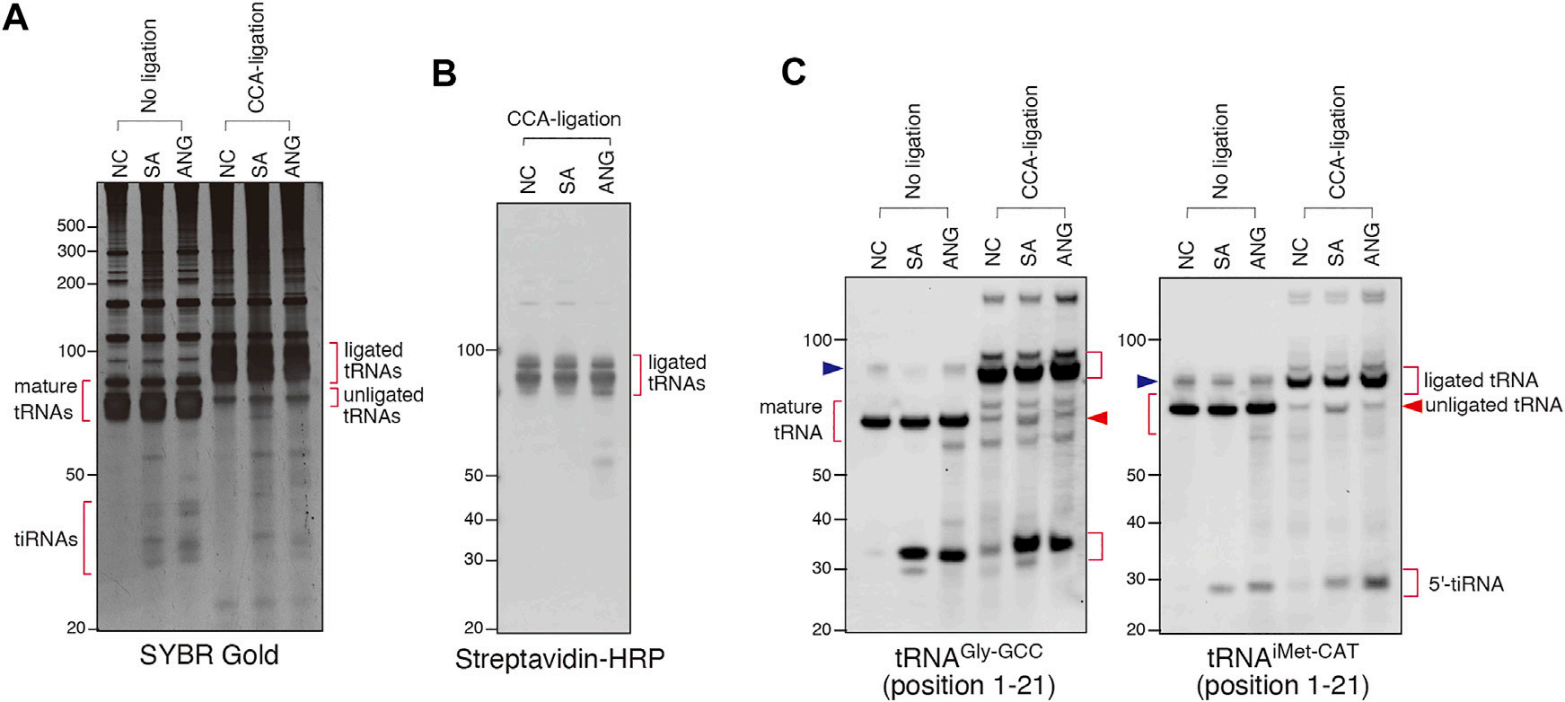
Sodium arsenite (SA) treatment induces CCA-deactivated tRNAs. **(A)** SYBR Gold staining of the CCA-specific ligation products. **(B)** Visualization of ligated tRNAs by streptavidin-biotin system. **(C)** Northern blot analysis showing that SA treatment slightly increases the amount of unligated tRNAs. The blue arrowheads indicate the bands for pre-tRNAs.

We used this approach to determine whether SA and/or ANG promote cleavage of the 3′-CCA termini *in cellulo* (Figure 4). We treated U2OS cells with SA or recombinant ANG, purified total RNA from the cells and proceeded with CCA-specific ligation using Rnl_2_ and ds-oligo containing biotin (“ligation 3″ in Figure 3A). RNA samples from all conditions were efficiently ligated as judged by SYBR Gold staining (Figure 4A, CCA-ligation) and immuno-blotting against biotin-containing oligo (Figure 4B). Figures 4A,B also showed that mature tRNAs were exclusively ligated, demonstrating excellent specificity of this method toward mature tRNAs. Using probes against specific tRNA species (tRNA^Gly-GCC^ and tRNA^iMet-CAT^, Figure 4C), we saw no difference in the amount of ligated tRNAs between control and ANG-treated samples. However, slight yet reproducible increase of unligated tRNAs was detected in the SA-induced samples (Figure 4C; Supplementary Figure S4, unligated tRNA). This unligated tRNAs in SA-induced samples seemed slightly shorter than those in NC and ANG groups, suggesting that SA treatment affected CCA cleavage. While the amount of unligated tRNA^Gly-GCC^ was slightly but significantly increased by SA treatment, that of unligated tRNA^iMet-CAT^ was not significantly increased (Supplementary Figure S4). These data suggest that even though SA treatment can induce CCA-deactivation, the proportion of CCA-deactivated tRNAs remains very low. It should also be noted that treatment of the purified total RNA with SA at various concentration *in vitro* did not trigger degradation, cleavage or shortening of tRNAs (Supplementary Figures S5A, S5B) thus rejecting the possibility that SA chemically triggers tRNA cleavage. Taken together, these data demonstrate that ANG does not efficiently cleave 3′-CCA termini of tRNAs *in cellulo*.

### CC-specific Ligation Shows That SA Promotes Cleavage of tRNAs’ CCA-Termini *in Cellulo*

**FIGURE 5:**
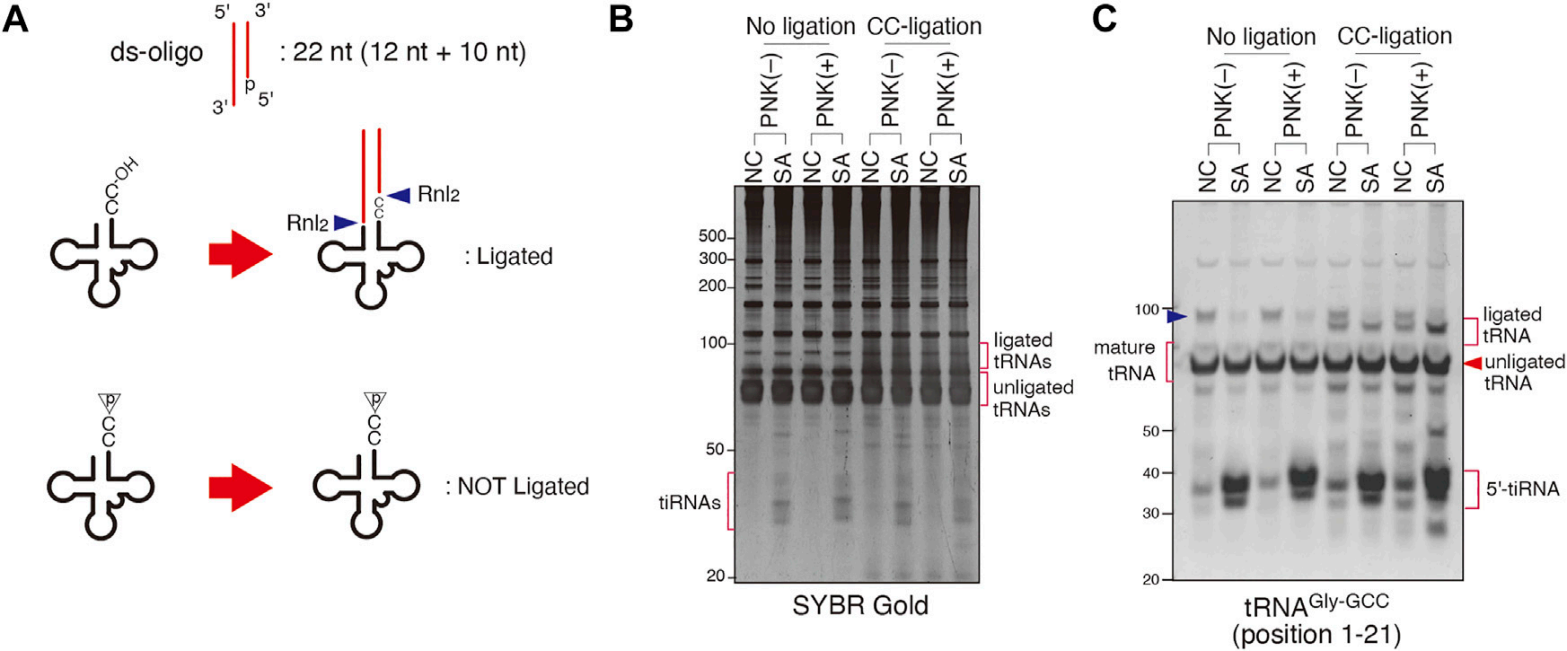
Sodium arsenite (SA) treatment induces CC-terminating tRNAs. **(A)** Schema for CC-specific ligation. Ligation products is generated only when CC-terminating tRNAs have hydroxyl residue on their 3′-end. **(B,C)** SA treatment increases CC-terminating tRNAs with 2′, 3′-cyclic phosphate residue. **(B)** SYBR Gold staining and **(C)** Northern blotting of CC-specific ligation products. The blue arrowhead indicates the band for pre-tRNA.

To further complement these findings, we have developed an alternative approach that aims on the ligation of only CC-terminating tRNAs, named “CC-specific ligation” (Figure 5A). In contrast to “CCA-specific ligation”, only CC-terminating tRNAs will be detected as ligation products by this method. Because ds-oligo has 5′-monophosphate and can only be ligated by Rnl_2_ to the CC-terminating tRNAs with 3′-hydroxyl group, if SA induces an RNase that cleaves tRNAs’ CCA termini to produce tRNAs terminating with CC-end with 2′, 3′-cyclic phosphate, such substrate will not be ligated (Figure 5A). To examine this possibility, we investigated the effect of pre-treatment of T4 PNK, which removes both a 3′-phosphate and 2′, 3′-cyclic phosphate to form a 3′-hydroxyl end, on CC-specific ligation products. As shown in Figures 5B,C, Rnl_2_ more efficiently ligates tRNAs from SA-treated cells that were pre-treated with T4 PNK. However, unless pre-treated with PNK, even tRNAs from SA-treated cells were not ligated to the oligo, suggesting that CC-terminating tRNAs contained 2′, 3′-cyclic phosphates at their 3′-ends. These findings indicate that an enzyme that triggers CCA cleavage under SA stress may belong to the RNase A superfamily members.

Interestingly, when total RNAs were subjected to *in vitro* ANG digestion, tRNAs were cleaved at both anticodon and 3′-CCA termini, and CCA-deactivated tRNAs were detected by CCA-specific ligation (Supplementary Figures S6A, S6B) and CC-specific ligation (Supplementary Figures S6C, S6D), which was in marked contrast to that from *in cellulo* ANG experiments (Figures 1, 4). These results clearly indicate that the specificity of ANG is significantly different between *in cellulo* and *in vitro* settings as previously reported (Akiyama et al., 2021).

Our data suggest that SA stress triggers the cleavage of CCA ends of tRNA *in cellulo* (Figures 4, 5). Although SA treatment readily results in oxidative stress, arsenic has multiple pleiotropic toxic effects by interacting various intracellular molecules [reviewed in (Shen et al., 2013)]. Treatment of cells with N-acetyl-cysteine (NAC), a potent antioxidant and reactive oxygen species scavenger (Aldini et al., 2018), did not prevent SA-induced CCA cleavage (Supplementary Figures S7A, S7B). Moreover, treatment with high dose of hydrogen peroxide (H_2_O_2_), a potent inducer of oxidative stress (Sies et al., 2017), also did not trigger CCA cleavage (Supplementary Figures S7C, S7D), in contrast to its specific effect on tRNA splicing resulting in the accumulation of 5′-leader-exon fragments of tRNA^Tyr−GTA^ (Hanada et al., 2013; Supplementary Figure S7D). These data suggest that oxidative stress is not a primarily trigger of SA-induced CCA cleavage.

### Involvement of Other, Besides ANG, RNases With Stress-Induced tRNA Cleavage

**FIGURE 6:**
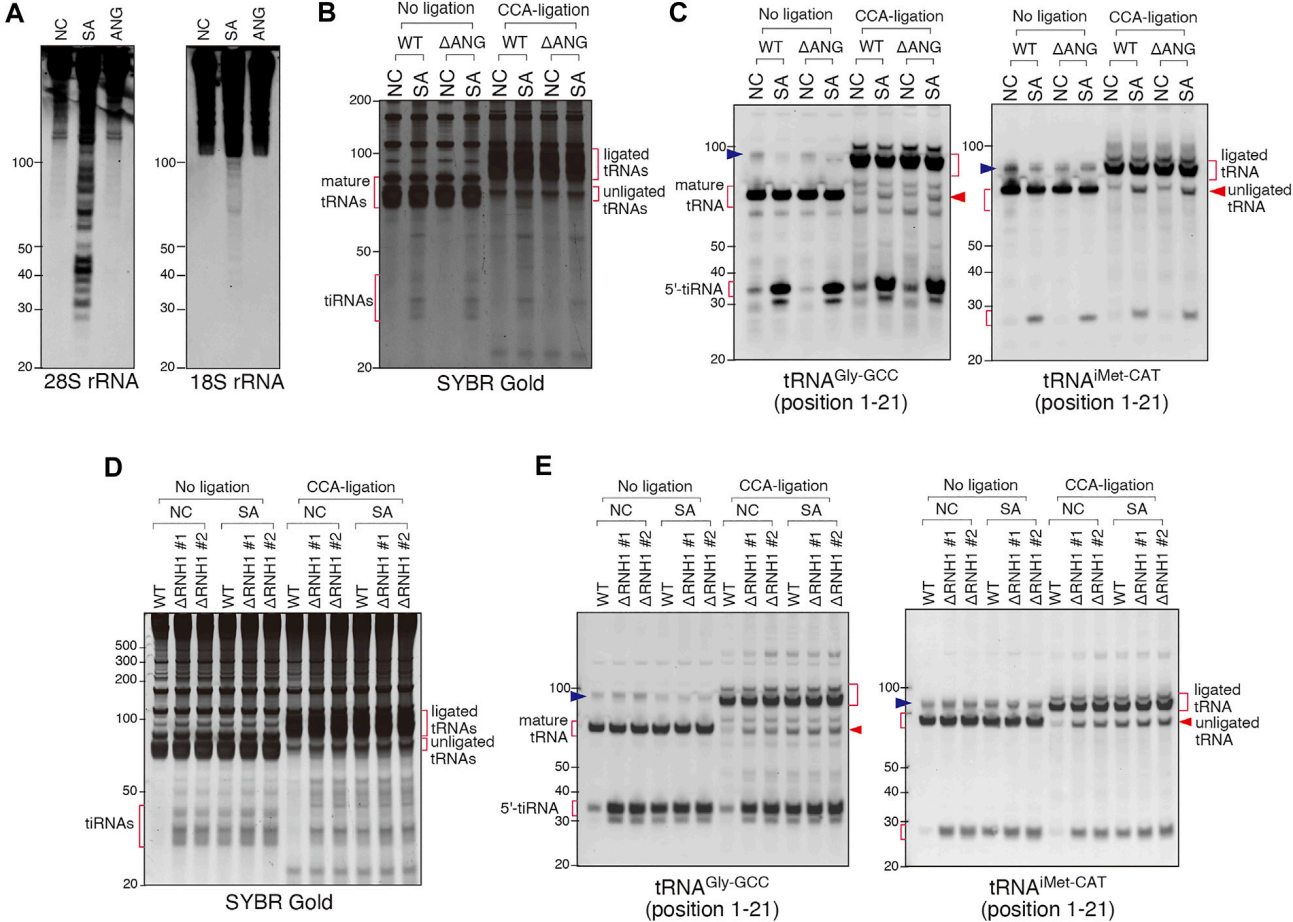
Involvement of other RNase A superfamily members than ANG in sodium arsenite (SA)-induced tRNA cleavage. **(A)** Northern blot analysis showing that SA treatment induces rRNA fragmentation but ANG does not. **(B,C)** SA treatment induces CCA-deactivated tRNAs even in ANG knockout (ΔANG) cells. **(B)** SYBR Gold staining and **(C)** Northern blotting of the CCA-specific ligation products. **(D,E)** RNH1 negatively regulates SA-induced tRNA cleavage. **(D)** SYBR Gold staining and **(E)** Northern blotting of the CCA-ligation products. Note that constitutive CCA-deactivation and tiRNA production are shown in RNH1 knockout (ΔRNH1) cells, and the amount of them was not increased by SA treatment. The blue arrowheads indicate the bands for pre-tRNAs.

In contrast to ANG treatment, SA treatment also triggered cleavage of other RNA substrates (e.g., rRNAs (Figure 6A), suggesting that SA activates other RNases. To determine the involvement of other RNase A superfamily members than ANG, we created a U2OS cell line variant with genetic knockout of ANG gene (ΔANG) (Supplementary Figures S1A–S1C). When WT and ΔANG cell lines were treated with SA, we observed no differences in the CCA cleavage as judged by the CCA-specific ligation of tRNA^Gly-GCC^ and tRNA^iMet-CAT^ (Figures 6B,C), suggesting that in the absence of ANG, SA-induced CCA-deactivation occurs *in cellulo*. In parallel, we observed that SA also triggered production of tiRNAs in ΔANG U2OS cells, suggesting that other cellular RNases are capable of cleaving tRNAs in the absence of ANG. This is in contrast to siRNA knockdown data where ANG depletion significantly affects tiRNA production (discussed later).

RNH1 (Ribonuclease/Angiogenin inhibitor 1) binds and inhibits activities of any RNase belonging to the RNase A superfamily (Dickson et al., 2005). To determine whether a CCA-cleaving enzyme is a member of RNase A superfamily, we created two RNH1 knockout U2OS cell lines (ΔRNH1 #1 and #2) (Supplementary Figures S1D–S1H). As shown in Figure 6D (no ligation, NC), in contrast to WT U2OS cells, ΔRNH1 U2OS cell lines promote spontaneous formation of tiRNAs even in the absence of SA treatment. Importantly, under SA treatment, there was no further enhancement of tiRNA production in ΔRNH1 cells (Figures 6D,E, No ligation), suggesting that in the absence of RNH1, RNase A superfamily members are fully activated, and tRNA cleavage is “saturated”. In addition, these data also suggest that SA-induced tiRNA production is likely due to activation of RNase A superfamily members (due to dissociation of them from RNH1, in other words) under this condition. In addition, as judged by the CCA-specific ligation assay, in the absence of RNH1, the amount of unligated tRNAs in ΔRNH1 U2OS cells (in the absence and presence of SA) was similar to that in WT U2OS cells treated with SA (Figures 6D,E, CCA-ligation), which suggests that SA-induced CCA-deactivation is also likely due to RNase A superfamily members, although it is not clear whether RNH1 may regulate other RNase A-unrelated RNases. Because SA treatment did not induce obvious degradation of RNH1 (Supplementary Figure S8A) or marked up-regulation of RNases (Supplementary Figure S8B), we suggest that SA-induced tRNA cleavage is likely due to inhibition of RNH1 function to inactivate RNase A superfamily enzymes.

## Discussion

In this study, we tested whether ANG cleaves the 3′-CCA termini of tRNAs *in cellulo*. If ANG efficiently cleaves 3′-CCA termini *in vivo*, it could contribute to stress-induced translational repression by mechanisms distinct from the reported before, where tRNA fragments directly interact with translational machinery (Ivanov et al., 2011; Sobala and Hutvagner 2013; Ivanov et al., 2014; Lyons et al., 2017; Mleczko et al., 2018; Fricker et al., 2019). However, our data using RNA-seq reveal that ANG does not affect the percentage of CCA- or CC-terminating species in tRNA fractions, thus demonstrating that ANG does not efficiently cleave 3′-CCA termini *in cellulo* (Figure 1B; Supplementary Table S3). In addition, we show that more than 98% of 3′-tiRNAs have 3′-CCA after ANG treatment, a percentage that exceed the 90.22% observed under control conditions (Figure 1C; Supplementary Table S3B), which is consistent with the previous report (Su et al., 2019). These RNA-seq based data suggest that ANG cleaves CCA termini of tiRNAs to a minimal extent and that it does not precede cleavage at the anticodon loop as suggested by *in vitro* studies (Czech et al., 2013).

We acknowledge limitations to our RNA-seq study. First, in our RNA-seq, we generated the libraries from the tRNA fraction (50–110 nt) or tiRNA fraction (20–50 nt) independently in order to generate enough depth of reads. Therefore, we cannot directly compare the results between the tRNA and tiRNA fractions. For example, we cannot directly analyze the proportion of tRNAs that were cleaved by ANG, or the ratio of anticodon cleavage to 3′-CCA cleavage by ANG. Second, because we defined the reads that overlapped the 3′-ends of tRNA genes as “3′-end containing reads” (Supplementary Figure S2C), some irregular reads could be included such as fragments which had 3′-trailer sequences (e.g., the read with ID:008 in Supplementary Figure S2C). Such irregular reads could contribute to the decrease in %CCA because they would be categorized as “others”. Because most of the 3′-tiRNAs and tRNAs had CCA at their 3′-termini and were categorized as “CCA-added” (Supplementary Figure S2C), such irregular fragments were estimated to be rare.

We also validated the ANG- or SA-mediated cleavage of 3′-CCA termini using two complementary biochemical approaches (Figures 3–5). The first approach, named “CCA-specific ligation”, is the method where only 3′-CCA termini-intact tRNAs are ligated to the oligo. It should be noted that CCA-deactivated tRNAs would be detected as unligated tRNAs in this method. In order to obtain the best result, we first optimized the condition of ligase reaction (Figure 3). First, we determined that the ligation efficiency of Dnl was much lower than that of Rnl_2_ (Figures 3B,C). Because Dnl had very low ligation efficiency, neither ligated tRNAs nor unligated tRNAs seemed to reflect CCA-integrity of tRNAs, because not all CCA-intact tRNAs is ligated, and substantial amount of tRNAs is unreacted and remains as unligated tRNAs (Figure 3B). We also determined that ds-oligo showed better ligation activity than hairpin oligo (Figure 3). For the ligation between tRNAs and hairpin oligo, the oligo must form hairpin structure. The lower ligation efficiency of hairpin oligo may be because it is less stable than ds-oligo, resulting that some proportion of the oligo cannot keep the hairpin structure. Using the optimized condition, we showed that there is no difference between ligation efficiency of untreated and ANG-treated tRNAs. However, we detected a slight (yet reproducible) increase in unligated tRNA signal in the case of SA-treated cells (Figure 4C). Importantly, unligated tRNAs in SA-treated cells were slightly shorter than those in untreated and ANG-treated tRNAs, which strongly suggests 3′-CCA cleavage by SA treatment. In order to confirm 3′-CCA cleavage by SA, we next developed the second ligation approach based on the ligation specific to CC-terminating tRNAs with 3′-hydroxyl group, named “CC-specific ligation”. In this method, if an RNase cleaves tRNAs to produce variants terminating with CC-end with 2′, 3′-cyclic phosphate, such tRNA substrate will not be ligated (Figure 5A). It should be noted that this “CC-specific ligation” method can distinguish CC-terminating tRNAs from other CCA-deactivated tRNAs such as C-terminating and CCA-less tRNAs. Again, and in agreement with other data, ligation of tRNAs from control and SA-treated cells shows that majority of tRNA species are CCA-terminating. Only slight increase in the population of 3′-CC terminating tRNAs with cyclic 2′, 3′-cyclic phosphate was observed by SA treatment (Figures 5B,C). Taken together, although SA treatment induces CCA-deactivation of tRNAs as previously reported (Czech et al., 2013), the proportion of CCA-deactivated tRNAs is very low. Therefore, the impact of CCA-deactivation on translation is estimated to be negligible.

It was also suggested that ANG-induced 3′-CCA cleavage may be reversible via repair of the cleaved CCA-ends by the CCA-adding enzyme TRNT1 (Czech et al., 2013). It should be noted that TRNT1 would need to resolve the 2′, 3′-cyclic phosphate to a 3′-hydroxyl residue before repair, the activity that has not been attributed to enzyme. Theoretically, the 2′, 3′-cyclic nucleotide 3′-phosphodiesterase (CNPase) could be involved with this reaction. However, although it has been suggested that CNPase is involved with the metabolism of endogenous 2′, 3′-cAMP especially in the myelin sheath (Verrier et al., 2012), the involvement of the enzyme in tRNA metabolism including tRNA splicing has not been established in the vertebrates (Myllykoski et al., 2016). Recent advances in RNA-seq methodologies have revealed that there exists a nonnegligible amount of RNA fragments possessing 2′, 3′-cyclic phosphate in the cell, especially under stress condition (Shigematsu et al., 2019; Shigematsu and Kirino 2020), suggesting that 2′, 3′-cyclic phosphate-containing RNAs are not easily catalyzed into 3′-hydroxyl forms once generated. Our data showed that efficient depletion of TRNT1 (Figure 2A) does not change percentage of CCA- and CC- terminating tiRNAs derived from cytosolic tRNAs in cells treated with recombinant ANG (Figure 2D and Supplementary Table S4), suggesting that TRNT1 does not play a role in regulating ANG-dependent tRNA cleavage. Although ANG did not cleave 3′-CCA termini, our biochemical experiments show that SA induces CCA-deactivation as previously reported (Czech et al., 2013). However, our CC-specific ligation method showed that SA-induced CCA-deactivated tRNAs possess 2′, 3′-cyclic phosphate residue (Figure 5), suggesting that TRNT1 cannot repair CCA-deactivated tRNAs under this condition. Further investigation is required to clarify the fate of CCA-deactivated tRNAs.

Finally, experiments using genetic knockouts of ANG and RNH1 suggest that: 1) SA-induced CCA cleavage is independent of ANG (Figures 6B,C); 2) RNH1 regulates SA-induced CCA cleavage (Figures 6D,E) suggesting that an unidentified RNase or RNases may be members of the RNase A superfamily; 3) RNH1 negatively regulates production of tiRNAs (Figures 6D,E); 4) In the absence of ANG, other stress-induced ribonuclease(s) can generate tiRNAs (Figures 6B,C). The latter observation is consistent with the recent publication from the Dutta lab that also showed that tiRNAs can also be generated by unknown RNase(s) in the conditions of high concentration SA-induced stress (Su et al., 2019). Although their data suggest that overexpression of ANG leads to cleavage of specific tRNA subset based on RNA-seq approach, our data suggest that treatment with recombinant ANG leads to production of 5′-tiRNAs from all tRNAs (Supplementary Figure S3). Nonetheless, both studies and our previous work (Saikia et al., 2012) agree that ANG is a stress-responsive RNase capable of tiRNA production through anticodon targeting but also other RNases can play a role in stress-induced tRNA cleavage. So far, we have not succeeded in identifying the RNase responsible for ANG-independent tRNA cleavage under stress condition in U2OS cells, because of very low expression level of examined RNases in U2OS cells (Supplementary Figure S8C). It can also be that in other cell types, other RNases may play dominant roles in tiRNA production when ANG is naturally not expressed or low expressed. However, our findings suggest that dissociation of RNase A superfamily members from RNH1 (or may be other unknown RNases interacting with RNH1) is the main mechanism for SA-induced tRNA cleavage (Figures 6D,E). In osteosarcoma cells, ANG seems to be a main RNase responsible for tiRNA production (Yamasaki et al., 2009). Interestingly, in the artificial conditions where ANG is genetically deleted, other RNases compensate its absence, at least in terms of tiRNA production. This parallels our other findings where different effectors of SG-mediated stress response often back up each other, e.g., loss of the key SG protein G3BP1 is compensated by overexpression of G3BP2 protein (Kedersha et al., 2016).

It is clear that activities of RNases responsible for SA-induced tRNA cleavage depend on the profile and abundances of RNase A superfamily enzymes, and possibly other RNases, expressed in each cell. Although SA-induced tRNA cleavage can be explained solely by activation of RNase A superfamily members (Figures 6D,E), it can be that other stress responsive RNases participate in tRNA cleavage. SA promotes tRNA cleavage in oxidative stress-independent manner *in cellulo* (Supplementary Figure S7) suggesting that it may activate downstream signaling pathways to dissociate RNases from RNH1, e.g., by post-translational modifications such as phosphorylation [discussed in (Sarangdhar and Allam 2021)]. Future studies on stress-induced tRNA cleavage will determine details of biogenesis of various tRNA fragments and roles of various RNase A superfamily members and other RNases in this process.

## Data Availability

The datasets presented in this study can be found at NCBI SRA under the BioProject ID: PRJNA770135, PRJNA770144, and PRJNA770170.
