# Supplementary material for: Selective Cleavage at CCA Ends and Anticodon Loops of tRNAs by Stress-Induced RNases
Source: Front Mol Biosci. 2022 Mar 1;9:791094. doi: 10.3389/fmolb.2022.791094 (PMC8920990; doi:10.3389/fmolb.2022.791094)
Supplement: Supplementary file 1 [file DataSheet1.PDF]

A

WT GGAAGAGATGGTGATGGCCTGGGCGTTTTGTTGTTGGTCTTCG-TGCTGGGTCTGGGTCTGACCCCACCGACCCTGGCTCAGGA
ΔANG GGAAGAGATGGTGATGGCCTGGGCGTTTTGTTGTTGGTCTTCGTTGCTGGGTCTGGGTCTGACCCCACCGACCCTGGCTCAGGA
*** **

B

Wild Type ANG
MVMGLGVLLLVFVLGLGLTPPTLAQDNSRYTHFLTQHYDAKPQGRDDRYCESIM
RRRGLTSPCKDINTFIHGNKRSIKAICENKNGNPHRENLRISKSSFQVTTCKLHG
GSPWPPCQYRATAGFRNVVVACENGLPVHLDQSIFRRP*

ΔANG
MVMGLGVLLLVFVAGSGSDPTDPGSG*

C

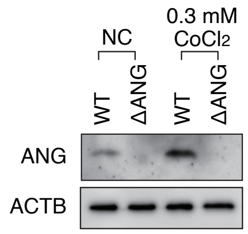

D

ΔRNH1 #1 Allele 1 CACCATGAGCCTGGACATCCAGAGCCTGGACATCCAGTTGTGAGGAGCTGAGCGACGCTAGATGGGCCGA
ΔRNH1 #1 Allele 2 CACCATGAGCCTGGACATCCAGAGC--------------------------------CTAGATGGGCCGA
RefSeq CACCATGAGCCTGGACATCCAGAGCCTGGACATCCAG-TGTGAGGAGCTGAGCGACGCTAGATGGGCCGA
************************* *************

E

Wild Type RNH1
MSLDIQSLDIQCEELSDARWAELLPLLQQCQVVRLDDCGLTEARCKDISSALRVNPALAELNLRSNELGDVGVHCVLQG
LQTPSCKIQKLSLQNCCLTGAGCGVLSSTLRTLPTLQELHLSDNLLGDAGLQLLCEGLLDPQCRLEKLQLEYCSLSAAS
CEPLASVLRAKPDFKELTVSNNDINEAGVRVLCQGLKDSPCQLEALKLESCGVTSDNCRDLCGIVASKASLRELALGSN
KLGDVGMAELCPGLLHPSSRLRTLWIWECGITAKGCGDLCRVLRAKESLKELSLAGNELGDEGARLLCETLLEPGCQLE
SLWVKSCSFTAACCSHFSSVLAQNRFLLELQISNNRLEDAGVRELCQGLGQPGSVLRVLWLADCDVSDSSCSSLAATLL
ANHSLRELDLSNNCLGDAGILQLVESVRQPGCLLEQLVLYDIYWSEEMEDRLQALEKDKPSLRVIS

ΔRNH1 #1 Allele 1
MSLDIQSLDIQL*

ΔRNH1 #1 Allele 2
MSLDIQS*

F

ΔRNH1 #2 CACCATGAGCCTGGACATCCAGAGC--------------------------------CTAGATGGGCCGA
RefSeq CACCATGAGCCTGGACATCCAGAGCCTGGACATCCAG-TGTGAGGAGCTGAGCGACGCTAGATGGGCCGA
************************* *************

G

ΔRNH1 #2
MSLDIQS*

H

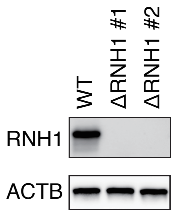

Supplementary Figure 1. Generation of ΔANG and ΔRNH1 cells. (A-C) Genotype of ΔANG cells. (A) Sequencing of ANG genomic locus. Initiator ATG is highlighted in green. (B) Predicted protein product. N-terminal signal peptide is underlined, and amino acids downstream of Cas9-induced insertion are highlighted in red. (C) Western blotting confirming the loss of ANG expression. Because baseline ANG expression level is very low, ANG knockout was also confirmed after $CoCl_2$ treatment (at 300 μM for 24h) to induce ANG expression. ACTB was used as a loading control. (D-H) Genotype of ΔRNH1 cells. (D) Sequencing of ΔRNH1 genomic locus in clone#1. Initiator ATG and terminal codon are highlighted in green and red, respectively. (E) Predicted protein product in ΔRNH1 clone#1 cells. (F) Sequencing of ΔRNH1 genomic locus in clone#2. (G) Predicted protein product in ΔRNH1 clone#2 cells. (G) Western blotting confirming the loss of RNH1 expression. ACTB was used as a loading control.

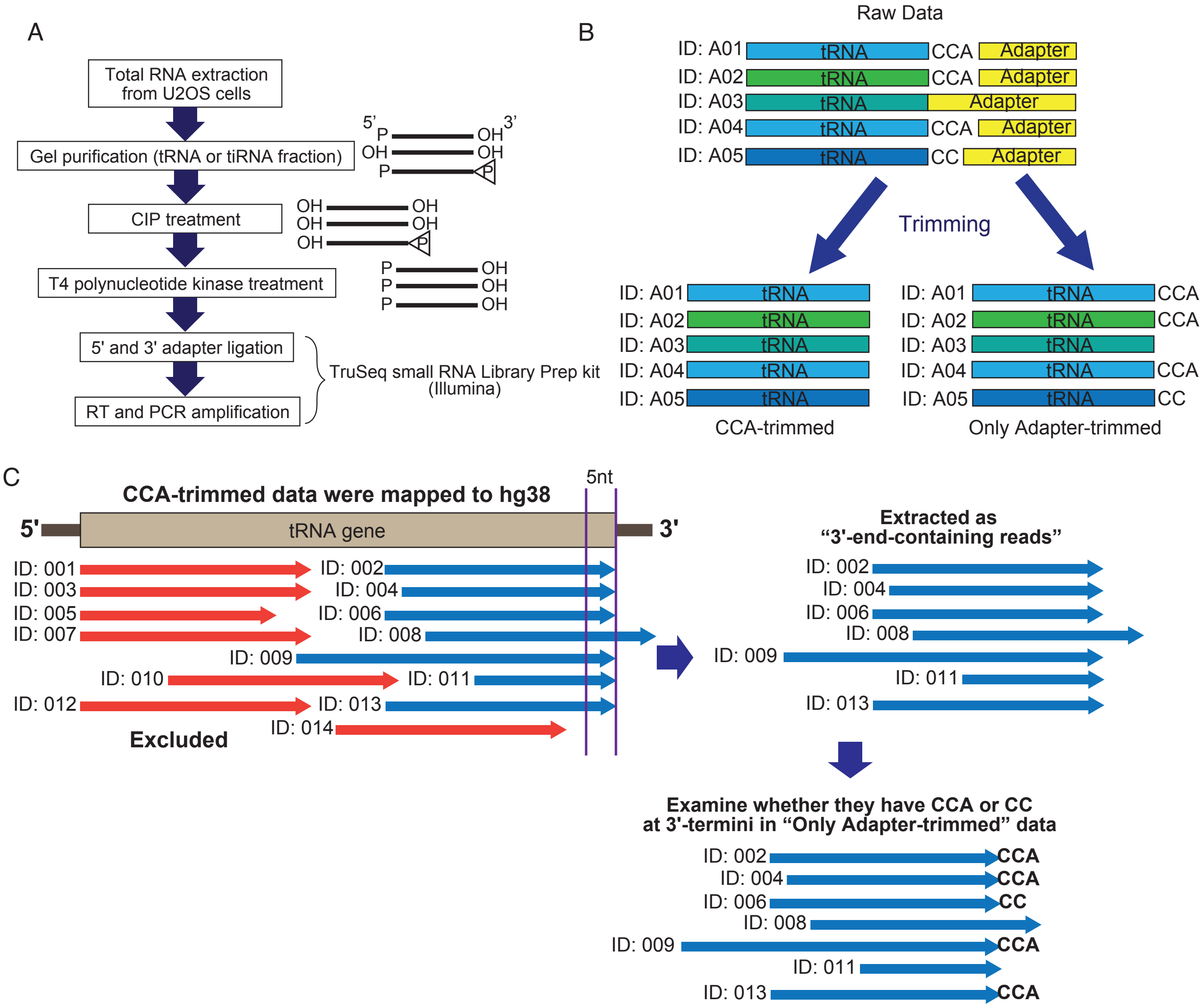

D

	tRNA genes ending with CCA	tRNA genes ending with (C)CC	the other tRNA genes	total
	109 genes	22 genes	500 genes	631 genes
CCA-added	A: ending with "CCACCA"	D: ending with "CCCCCA"	G: ending with "CCA"	A+D+G
CC-terminating	B: ending with "CCACC"	E: ending with "CCCCC"	H: ending with "CC"	B+E+H
the others	X-(A+B)	Y-(D+E)	Z-(G+H)	X+Y+Z-(A+B+D+E +G+H)
total counts	X	Y	Z	X+Y+Z

Supplementary Figure 2. Workflow for preparation of RNA sequencing library. (A) Flowchart of the preparation of the library. OH: hydroxyl group, P: 5'-phosphate, P in the triangle: 2', 3'-cyclic phosphate. (B) Diagram of trimming for the calculation of CCA - or CC-terminating reads. (C) Extraction of 3'-end containing reads and calculation of the proportion of CCA- or CC-terminating reads. In this example, %CCA and %CC are 57.1% (4/7) and 14.3% (1/7), respectively. (D) Calculation of the proportion of CCA-added (%CCA) or CC-terminating (%CC) fragments. The proportions were calculated as follows: %CCA = 100 x (A+D+G) / (X+Y+Z), %CC = 100 x (B+E+H) / (X+Y+Z). Note that all 22 genes ending with CC have C before CC (i.e. all the genes end with "CCC"). Therefore, the mature tRNAs derived from these genes should end with CCCCCA.

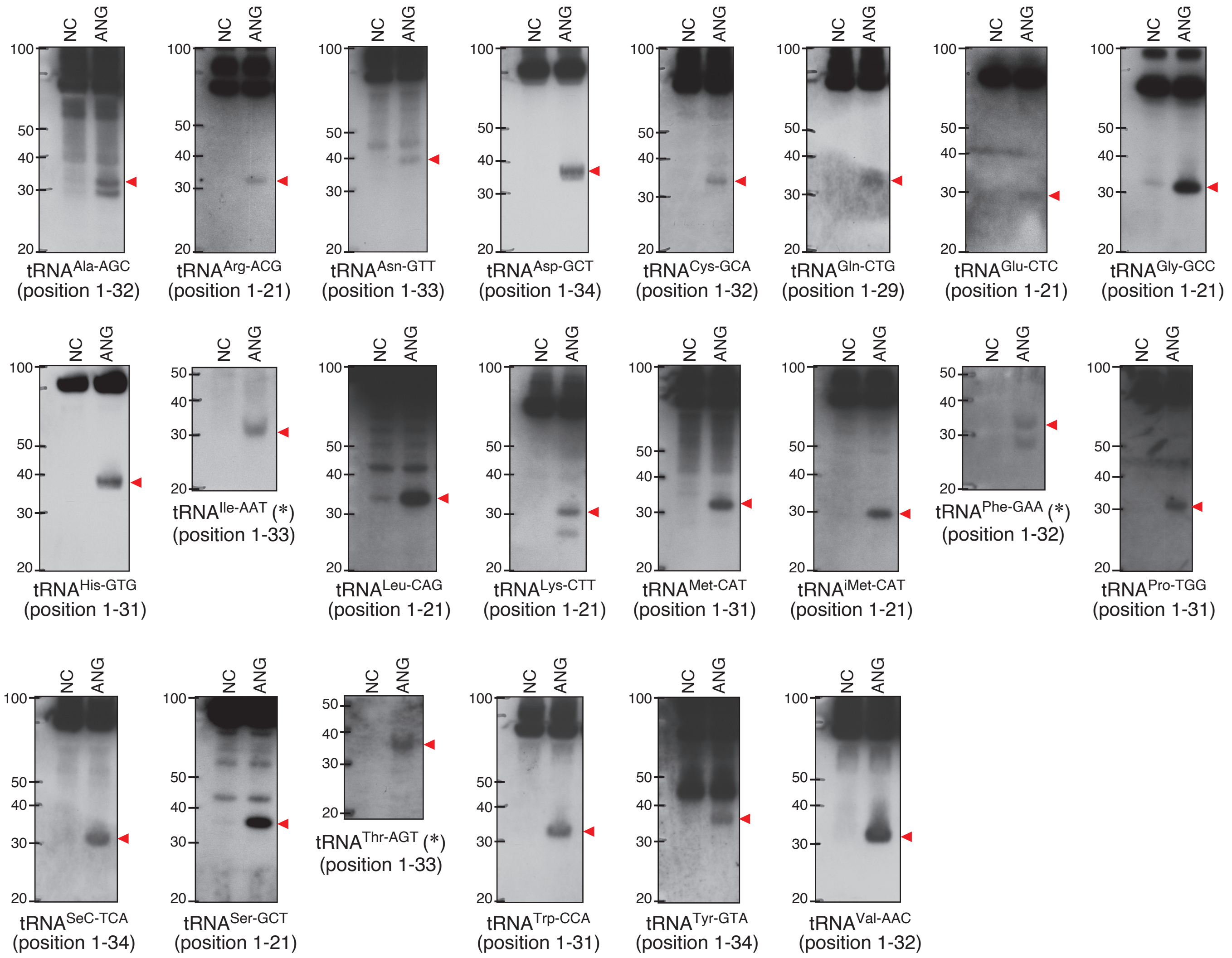

Supplementary Figure 3. ANG produces 5'-tiRNAs from all tRNAs. Northern blotting was performed for all kinds of tRNAs. (*): Chemical cross-linking was performed as described in Materials and Methods section to improve the sensitivity.

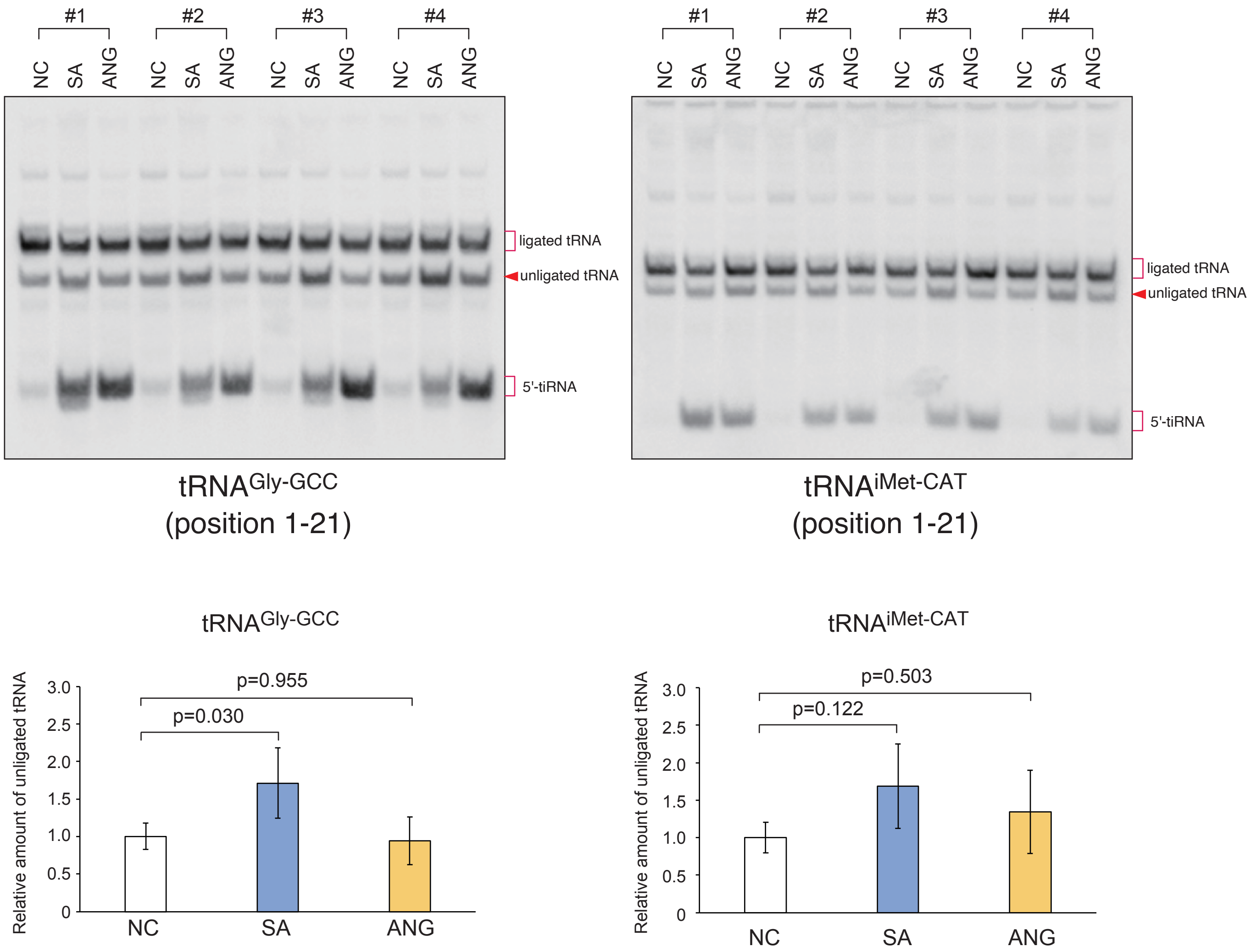

Supplementary Figure 4. Sodium arsenite-induced CCA-deactivation evaluated by CCA-specific ligation method. The intensities of the bands for unligated tRNA from 4 independent experiments were measured using Image J software and subjected to Dunnett's test.

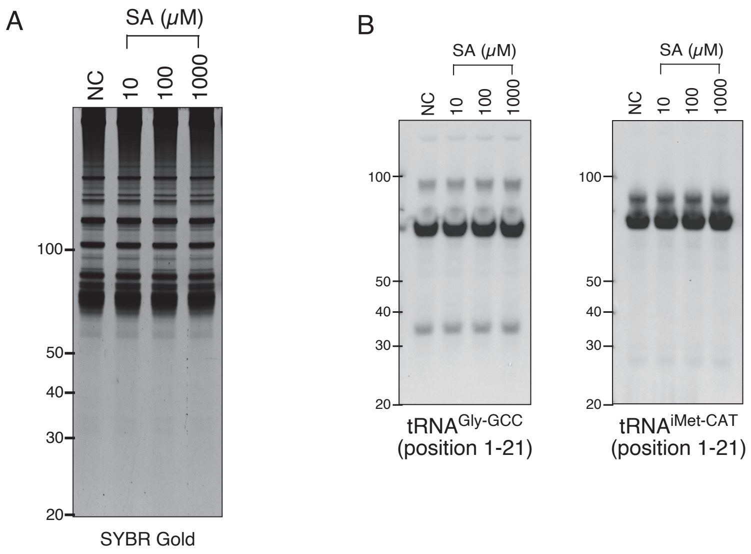

Supplementary Figure 5. Sodium arsenite does not induce any RNA degradation *in vitro*. Total RNAs from U2OS cells were incubated with sodium arsenite at the concentrations as indicated at room temperature for 1 hour. (A) SYBR Gold staining and (B) Northern blotting.

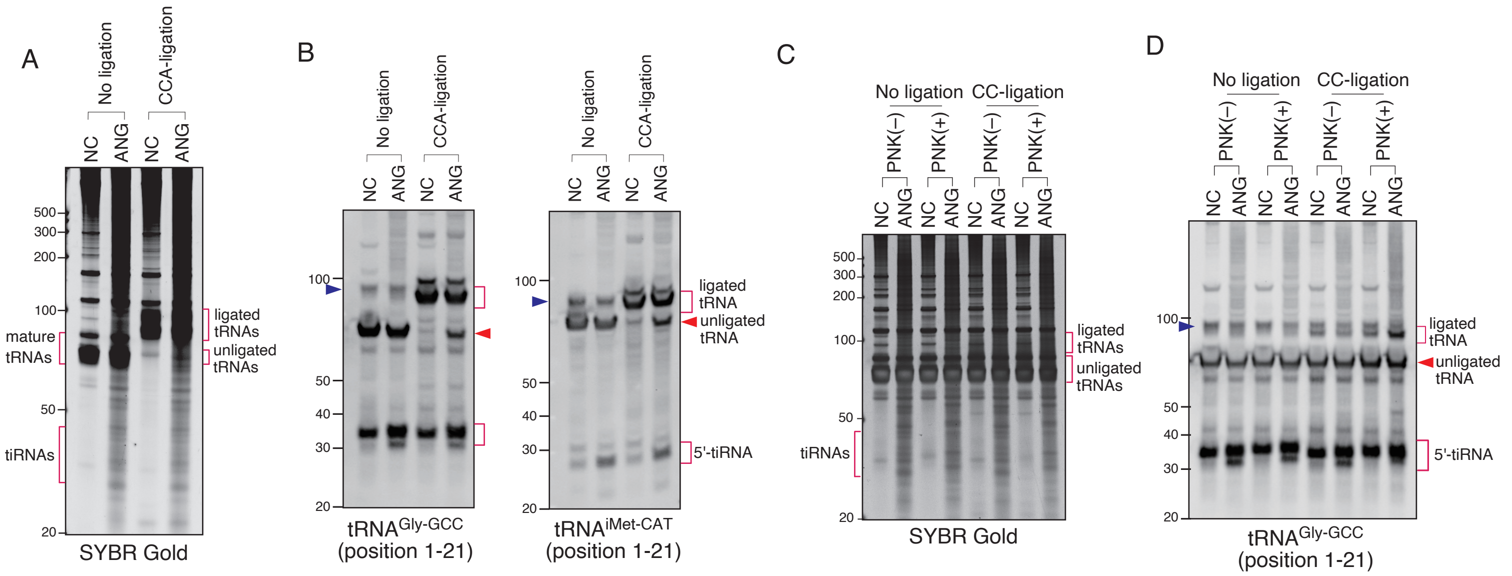

Supplementary Figure 6. *In vitro* ANG digestion produces CCA-deactivated tRNAs. Total RNAs were subjected to *in vitro* ANG digestion followed by CCA-specific or CC-specific ligation. (A-B) CCA-specific ligation. (A) SYBR Gold staining and (B) Northern blotting. (C-D) CC-specific ligation combined with PNK pretreatment. (C) SYBR Gold staining and (D) Northern blotting. The blue arrowheads indicate the bands for pre-tRNAs.

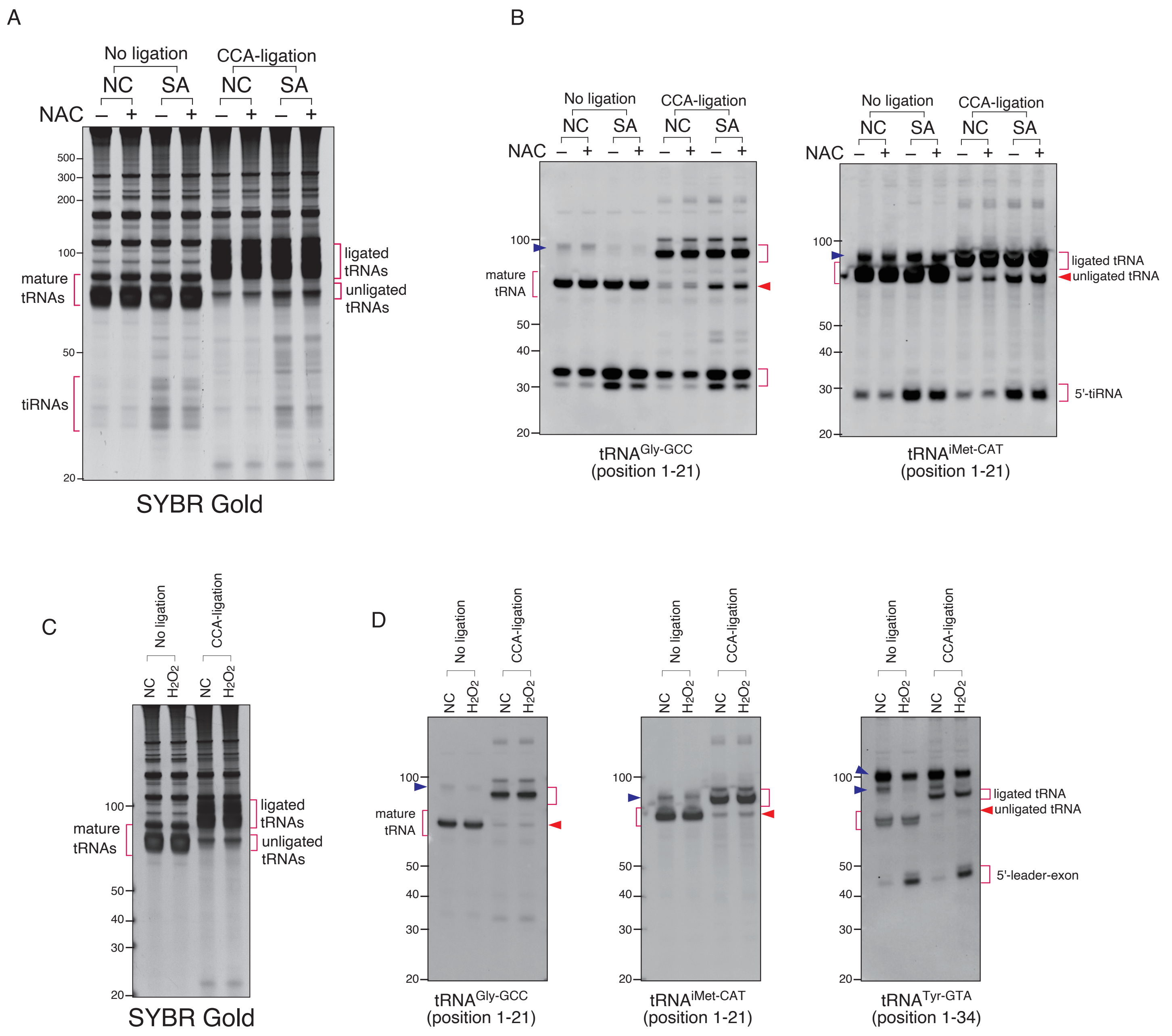

Supplementary Figure 7. Sodium arsenite-induced CCA-deactivation is independent of oxidative stress. (A-B) N-acetylcysteine (NAC) pre-treatment has no effect on sodium arsenite-induced CCA-deactivation. (A) SYBR Gold staining and (B) Northern blotting. U2OS cells were pre-treated with 20 mM NAC (pH 7.5) (Sigma-Aldrich) for 1 hr before sodium arsenite treatment, followed by RNA purification and CCA-specific ligation. NAC pre-treatment did not decrease the amount of CCA-deactivated tRNAs, in contrast to slight decrease of tiRNAs. (C-D) Hydrogen peroxide (H_2O_2) does not induce CCA-deactivation. (C) SYBR Gold staining and (D) Northern blotting. U2OS cells were treated with 500 μM of H_2O_2 for 1 hr, followed by RNA purification and CCA-specific ligation. H_2O_2 treatment did not induce either tiRNA production or CCA-deactivation. On the other hand, H_2O_2 treatment induced the generation of the 5'-leader-exon fragment derived from tRNA-Tyr-GTA as previously reported.

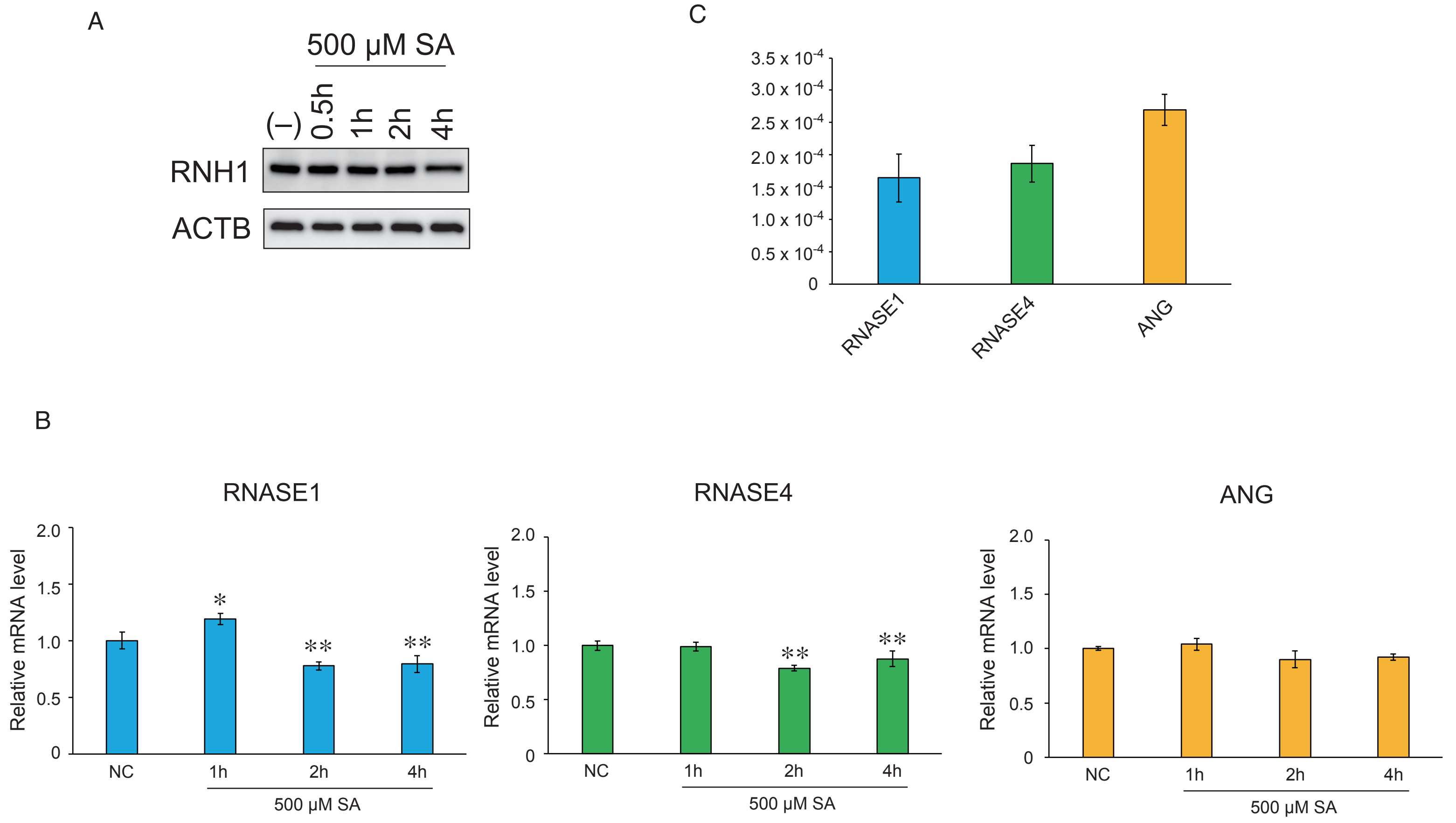

Supplementary Figure 8. Characterization of sodium arsenite treatment in U2OS cells. (A) Time course of RNH1 protein levels during SA treatment. ACTB was used as loading control. (B) Transcription levels of RNASE1, RNASE4 and ANG in U2OS cells during SA treatment. Relative mRNA expressions were normalized to GAPDH mRNA levels. (C) Baseline expression levels of RNASE1, RNASE4 and ANG in U2OS cells. Relative expression levels to GAPDH mRNA are shown.

Oligos		Sequence
Hairpin-oligo for CCA-specific ligation		/5Phos/cgcacugcTTTTTGCAGTGCGTGGN
double-strand oligo for CCA-specific ligation	5'-oligo	ACTGGATACTGgn
	3'-oligo	/5Phos/GTATCCAGTT/3Bio/
double-strand oligo for CC-specific ligation	5'-oligo	ACTGGATACggn
	3'-oligo	/5Phos/GTATCCAGTT

Supplementary Table 1. The sequences of oligos for CCA-specific or CC-specific ligation method. Capital letters indicate DNA, and small letters indicate RNA. N: mix of A, T, G and C. n: mix of a, u, g and c. 5Phos: 5'-phosphorylated. 3Bio: 3'-biotinylated.

Probes	Sequences
tRNA$^{Ala-AGC}$ (position 1-32)	5'-AAGCACGCGCTCTACCACTGAGCTACACCCCC-3′
tRNA$^{Arg-ACG}$ (position 1-21)	5'-TATCCATTGCGCCACTGGCCC-3'
tRNA$^{Asn-GTT}$ (position 1-33)	5'-GCCGAACGCGCTAACCGATTGCGCCACAGAGAC-3'
tRNA$^{Asp-GTC}$ (position 1-34)	5'-CAGGCGGGGATACTCACCACTATACTAACGAGGA-3'
tRNA$^{Cys-GCA}$ (position 1-32)	5'-AGTCAAATGCTCTACCACTGAGCTATACCCCC-3'
tRNA$^{Gln-CTG}$ (position 1-29)	5'-CAGAGTGCTAACCATTACACCATGGAACC-3'
tRNA$^{Glu-CTC}$ (position 1-21)	5'-TAACCACTAGACCACCAGGGA-3'
tRNA$^{Gly-GCC}$ (position 1-21)	5'-CTACCACTGAACCACCCATGC-3'
tRNA$^{His-GTG}$ (position 1-31)	5'-CGCAGAGTACTAACCACTATACGATCACGGC-3'
tRNA$^{Ile-AAT}$ (position 1-33)	5'-GCACCACGCTCTAACCAACTGAGCTAACCGGCC-3'
tRNA$^{Leu-CAG}$ (position 1-21)	5'-AGACCGCTCGGCCATCCTGAC-3'
tRNA$^{Lys-CTT}$ (position 1-21)	5'-TACCGACTGAGCTAGCCGGGC-3'
tRNA$^{Met-CAT}$ (position 1-31)	5'-ACTGACGCGCTACCTACTGCGCTAACGAGGC-3'
tRNA$^{iMet-CAT}$ (position 1-21)	5'-CTTCCGCTGCGCCACTCTGCT-3'
tRNA$^{Phe-GAA}$ (position 1-32)	5'-GTCTAACGCTCTCCCAACTGAGCTATTTCGGC-3'
tRNA$^{Pro-TGG}$ (position 1-21)	5'-ATACCCCTAGACCAACGAGCC-3'
tRNA$^{SeC-TCA}$ (position 1-34)	5'-GCCTGCACCCCAGACCACTGAGGATCATCCGGGC-3'
tRNA$^{Ser-GCT}$ (position 1-21)	5'-TAACCACTCGGCCACCTCGTC-3'
tRNA$^{Thr-AGT}$ (position 1-33)	5'-GACAGGCGCTTTAACCAACTAAGCCACGGCGCC-3'
tRNA$^{Trp-CCA}$ (position 1-31)	5'-GTCAGACGCGCTGCCGTTGCGCCACGAGGTC-3'
tRNA$^{Tyr-GTA}$ (position 1-34)	5'-CAGTCCTCCGCTCTACCAACTGAGCTATCGAAGG-3'
tRNA$^{Val-AAC}$ (position 1-32)	5'-GGCGAACGTGATAACCACTACACTACGGAAAC-3'
28S rRNA	5'-GGGTGAACAATCCAACGCTTGGTG-3'
18S rRNA	5'-AGAGGAGCGAGCGACCAAAGGAA-3'

Supplementary Table 2. Sequences of DNA-oligo probes for Northern blotting.

A

tRNA-fraction	Control	ANG	P value
total fragments	188,990 ± 7,551	164,810 ± 3,691	0.0452
CCA-added	179,947 ± 7,085	156,044 ± 3,600	0.0396
CC-terminating	1,170 ± 163	1,020 ± 89	0.4663
the others	7,873 ± 524	7,745 ± 451	0.8623
%CCA-added	95.22 ± 0.22	94.68 ± 0.22	0.1578
%CC-terminating	0.61 ± 0.06	0.62 ± 0.05	0.9433

B

tiRNA-fraction	Control	ANG	P value
total fragments	38,375 ± 2,787	240,173 ± 27,604	0.0019
CCA-added	34,621 ± 2,539	236,797 ± 27,353	0.0018
CC-terminating	1,151 ± 301	1,765 ± 270	0.2039
the others	2,602 ± 410	1,161 ± 12	0.0729
%CCA-added	90.22 ± 0.81	98.58 ± 0.08	0.0005
%CC-terminating	2.93 ± 0.55	0.73 ± 0.05	0.0166

Supplementary Table 3. Effect of ANG treatment on CCA-termini of (A) tRNAs and (B) 3'-tiRNA. Values are expressed as means ± SE (n=3).

A

tiRNA fraction of mito-tRNA-Ser-GCT	NC		ANG	
	siControl	siTRNT1	siControl	siTRNT1
total fragments	59.0 ± 10.0	38.4 ± 3.5	21.4 ± 4.0	7.2 ± 0.6
CCA-added	36.6 ± 7.4	17.5 ± 1.9*	13.2 ± 2.5	2.8 ± 0.5
CC-terminating	11.0 ± 0.8	9.3 ± 0.2	4.0 ± 0.8	2.3 ± 0.3
the others	11.4 ± 2.2	11.6 ± 1.6	4.2 ± 1.1	2.0 ± 0.1
%CCA-added	61.4 ± 2.0	45.5 ± 1.7**	61.5 ± 6.7	39.0 ± 1.0††
%CC-terminating	19.4 ± 2.5	24.5 ± 1.7	19.1 ± 2.6	32.1 ± 1.5†

B

tiRNA fraction	NC		ANG	
	siControl	siTRNT1	siControl	siTRNT1
total fragments	22,306 ± 628	25,342 ± 1,176	124,504 ± 16,783**	109,038 ± 5,698‡‡
CCA-added	19,728 ± 529	22,233 ± 1,119	122,073 ± 16,579**	106,604 ± 5,531‡‡
CC-terminating	664 ± 22	796 ± 43	934 ± 83	860 ± 89
the others	1,913 ± 96	2,313 ± 109	1,496 ± 95	1,574 ± 91‡‡
%CCA-added	88.45 ± 0.26	87.70 ± 0.58	98.98 ± 0.27**	97.77 ± 0.06‡‡
%CC-terminating	2.98 ± 0.01	3.15 ± 0.23	0.77 ± 0.10**	0.79 ± 0.05‡‡

Supplementary Table 4. TRNT1 does not affect the proportion of CCA-added 3'-tiRNAs in ANG-treated cells. (A) Effect of TRNT1 knockdown on the proportion of CCA-added 3'-tiRNAs derived from mitochondrial tRNA-Ser-GCT. Values are expressed as means ± SE (n=3). *: $p<0.05$ VS siControl-NC, ** $p<0.01$ VS siControl-NC, †: $p<0.01$ VS siTRNT1-NC, ††: $p<0.01$ VS siTRNT1-NC. (B) Effect of TRNT1 knockdown on the proportion of CCA-added and CC-added 3'-tiRNAs in ANG-treated cells. Values are means ± SE (n=3). **: $p<0.01$ VS siControl-NC, ‡‡: $p<0.01$ VS siControl-ANG.
